# Generation and characterization of novel recombinant anti-hERG1 scFv antibodies for cancer molecular imaging

**DOI:** 10.18632/oncotarget.26200

**Published:** 2018-10-09

**Authors:** Claudia Duranti, Laura Carraresi, Angelica Sette, Matteo Stefanini, Tiziano Lottini, Silvia Crescioli, Olivia Crociani, Luisa Iamele, Hugo De Jonge, Ermanno Gherardi, Annarosa Arcangeli

**Affiliations:** ^1^ Department of Experimental and Clinical Medicine, Section of Internal Medicine, University of Florence, Florence, Italy; ^2^ DIVAL Toscana Srl, Sesto Fiorentino, Florence, Italy; ^3^ Department of Molecular Medicine, University of Pavia, Pavia, Italy; ^4^ Present address: Crescendo Biologics Ltd., Babraham Research Campus, Cambridge, UK; ^5^ Present address: Department of Dermatology, King’s College, London, UK

**Keywords:** cancer diagnostics, molecular imaging, hERG1 potassium channels, antibody engineering

## Abstract

Modern molecular imaging techniques have greatly improved tumor detection and post-treatment follow-up of cancer patients. In this context, antibody-based imaging is rapidly becoming the gold standard, since it combines the unique specificity of antibodies with the sensitivity of the different imaging technologies. The aim of this study was to generate and characterize antibodies in single chain Fragment variable (scFv) format directed to an emerging cancer biomarker, the *human ether-à-go-go-related gene-1* (hERG1) potassium channel, and to obtain a proof of concept for their potential use for *in vivo* molecular imaging.

The anti-hERG1scFv was generated from a full length monoclonal antibody and then mutagenized, substituting a Phenylalanine residue in the third framework of the V_H_ domain with a Cysteine residue. The resulting scFv-hERG1-Cys showed much higher stability and protein yield, increased affinity and more advantageous binding kinetics, compared to the “native” anti-hERG1scFv. The scFv-hERG1-Cys was hence chosen and characterized: it showed a good binding to the native hERG1 antigen expressed on cells, was stable in serum and displayed a fast pharmacokinetic profile once injected intravenously in nude mice. The calculated half-life was 3.1 hours and no general toxicity or cardiac toxic effects were detected. Finally, the *in vivo* distribution of an Alexa Fluor 750 conjugated scFv-hERG1-Cys was evaluated both in healthy and tumor-bearing nude mice, showing a good tumor-to-organ ratio, ideal for visualizing hERG1-expressing tumor masses *in vivo*.

In conclusion, the scFv-hERG1-Cys possesses features which make it a suitable tool for application in cancer molecular imaging.

## INTRODUCTION

Molecular imaging techniques are continuously progressing, allowing non-invasive *in vivo* visualization of biological and pathological processes at the molecular and cellular levels. These methods are particularly suitable for application in oncology, where the improvement of available technologies has been paralleled by the development of targeting agents that can assure the high selectivity necessary for appropriate cancer diagnostics. Antibody-based imaging is becoming the gold standard in this field, since the combination of different imaging methodologies with tumor specificity has the potential to greatly improve cancer diagnosis and therapy follow up [1–5]. However, intact monoclonal antibodies (mAbs) are large (150 kDa) molecules, with generally slow pharmacokinetics, slow blood clearance, sub optimal tumor penetration and accumulation, and are often difficult to produce [6]. These characteristics can delay the time point for imaging, and often results in sub-optimal contrast between the tumor mass and the surrounding normal tissue. Antibodies in the single chain Fragment variable (scFv) format seem the optimal candidates to overcome such hindrances. A scFv antibody consists of variable regions of heavy (V_H_) and light (V_L_) chains (i.e. the smallest unit of an immunoglobulin molecule which binds its specific antigen), joined together by a flexible peptide linker. The reduced size (usually 25–30 kDa) of scFv antibodies speeds up their penetration into tissues and enhances their clearance from the blood, thus making scFv antibodies preferred tools for *in vivo* diagnostic purposes [7]. Moreover, scFv antibodies can be often easily expressed in functional form in *E. coli* or yeasts, facilitating protein engineering to further increase the affinity or modify the specificity [8]. Finally, it is possible to conjugate the recombinant proteins with quantum dot, fluorescent dyes or other moieties, hence potentiating their exploitability for *in vivo* imaging. For these reasons, scFv antibodies are becoming the ideal candidates in imaging applications, especially for cancer diagnostics [2–5].

The huge amount of work aimed at the identification of novel cancer biomarkers suitable for both therapeutic and diagnostic purposes, has recently led to consider plasma membrane proteins devoted to ion transport as good candidates for targeting cancer cells both *in vitro* and *in vivo* [9]. Indeed, ion channels and transporters are frequently over-expressed in cancers cells, hence behaving as novel cancer associated antigens. What is more, ion channels and transporters are easily accessible due to their expression on the plasma membrane [10]. Although technically challenging due to the limited availability of antigenic extracellular epitopes in this class of membrane proteins [11], both full length and scFv antibodies have been recently developed against those ion channels and transporters over- or mis-expressed in human cancers. For example, a scFv against Kv 10.1 (EAG1), a member of the *ether-à-go-go* (EAG) family of potassium channels frequently expressed in human cancers, has been raised and fused to the human soluble tumor necrosis factor-related apoptosis-inducing ligand (TRAIL), showing a selective pro-apoptotic activity on Kv 10.1 positive cancer cells [12]. Another member of the same family, Kv 11.1 (hERG1), besides being highly expressed in human cardiac myocytes, where it constitutes the molecular correlate of the repolarizing current IKr, is often over-expressed in human cancers [13]. In cancer, hERG1 regulates different aspects of neoplastic progression (i.e. cell proliferation and survival, cell invasiveness and angiogenesis), and behaves as a powerful diagnostic and prognostic marker both in solid cancers [14, 15] and hematologic malignancies [16, 17]. However, due to the possible cardiac side effects (lengthening of the electrocardiographic (ECG) QT interval and triggering of ventricular arrhythmia) that hERG1 blockade may produce, this channel is generally considered an undesirable pharmacologic target [14, 18]. Therefore, many precautions must be taken when designing tools to target hERG1 in cancer, in order to avoid potentially harmful cardiac effects. To recognize hERG1 in normal and cancer tissues, we developed a unique anti-hERG1 monoclonal antibody (hERG1-mAb) which recognizes an extracellular epitope (the S5-P loop) of the channel protein and hence can be used both on live and fixed cells without permeabilization [19]. However, the hERG1-mAb cannot be applied for *in vivo* cancer imaging, due to its large dimension and long half-life [20], which could impair tumor penetration and, conversely, facilitate the binding to the heart, i.e. the tissue where hERG1 is physiologically expressed.

To exploit hERG1 as a novel target for *in vivo* molecular imaging, we generated and characterized smaller size antibody fragments in the scFv format, with the aim of obtaining the proof of concept for their further use as a molecular tool for *in vivo* cancer imaging.

## RESULTS

### Generation of the scFv-hERG1

**Figure 1:**
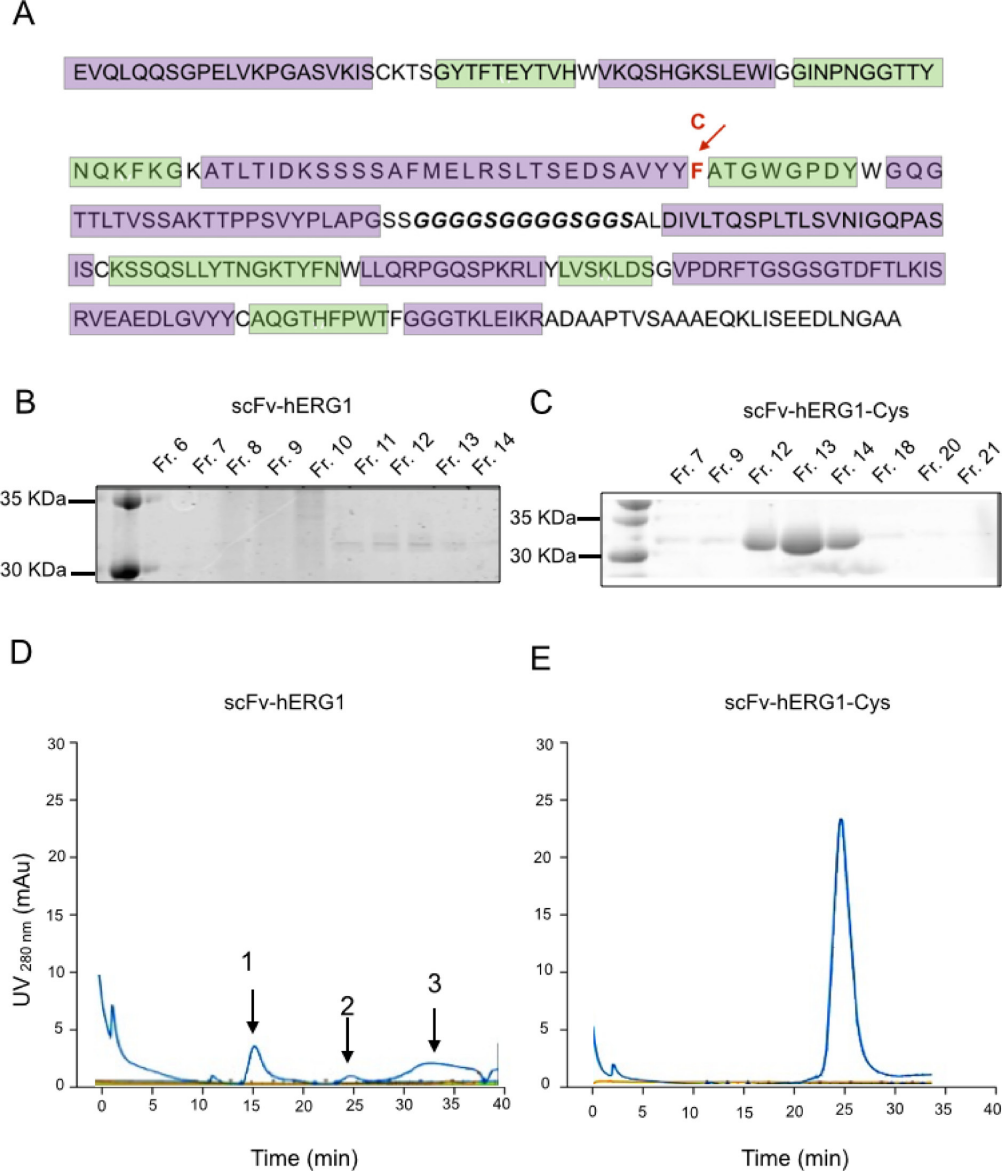
Biochemical characterization of scFv-hERG1 and scFv-hERG1-Cys antibodies (**A**) Amino acid sequence of the scFv-hERG1-encoding cassette. The CDRs of the heavy and light chain, identified according to the Kabat scheme through the ANARCI software, are highlighted in green; the glycine-serine linker region is in bold. The arrow indicates the F (Phenylalanine) amino acid in position 92 (red), which was replaced by a C (Cystein), as reported in the line above. SDS-PAGE and Coomassie Brilliant blue staining performed on different fractions (Fr.) obtained after AKTA purification of scFv-hERG1 (**B**) and scFv-hERG1-Cys (**C**). After dialysis using Slide-A-Lyzer™ Dialysis Cassettes (Thermo Fisher, Massachusetts, USA) against PBS, scFv protein absorbance was measured at 280 nm and the Lambert-Beer equation was applied for antibody quantification. Size-Exclusion Chromatography (SEC) of scFv-hERG1 (**D**) and scFv-hERG1-Cys (**E**). According to the column used (Superdex 75, Ge Healthcare), proteins with a molecular weight around 30 KDa, such as scFv, should have a retention time of roughly 24–25 min. For the scFV-hERG1, the first peak, arbitrarily labelled as 1, corresponds to a retention time of 15 min and represents the aggregated form of the antibody; the second peak, arbitrarily labelled as 2, corresponds to a retention time of 24 min, and represents the scFv antibody; the third peak, arbitrarily labelled as 3, corresponds to 30–35 min retention time, and represents the degraded form of the protein. For the scFv-hERG1-Cys, a single peak is visible with retention time of nearly 24 min, corresponding to what expected for a scFv molecule.

The scFv-hERG1 was generated cloning the two variable heavy (V_H_) and light (V_L_) domains, amplified from the hERG1-mAb into the pCR-Blunt vector, following the procedure described in [21]. The DNA obtained from several E. coli positive colonies was sequenced and the most common V_H_ and V_L_ sequences were chosen (see Materials and Methods). V_H_ and V_L_ were then sequentially cloned into the pHenIX phagemid, which contains the sequence for the peptide linker necessary to join the carboxyl terminus of V_H_ to the amino terminus of V_L_, and hence allows the proper assembly of the scFv-hERG1 encoding cassette. The nucleotide sequence of this cassette is in Supplementary Figure 1, whereas the resulting amino acid sequence, obtained through the ExPASy translation tool, is shown in Figure 1A. The scFv-hERG1 expression cassette was then moved from pHenIX into pPIC9K and used to transform the GS115 P. pastoris yeast strain through the spheroplasting technique. We then chose the clone (G3) which showed the highest expression level of the protein after 72 hours of culture (Supplementary Figure 2A and 2B). The scFv-hERG1 antibody purified from scaled up (one liter) cultures was analyzed through SDS-PAGE and Coomassie Brilliant Blue staining (Figure 1B). The concentration of pooled fractions (Fr.) 11–14 was 0.050 mg/ml, with a total amount of 100 μg. Size-exclusion chromatography (SEC) (Figure 1D) showed the presence of several peaks in the chromatogram: in particular, peaks 1 and 3 indicated the presence of aggregated and degraded proteins, respectively, in addition to the monomeric form, witnessed by peak 2 (see the gel in Supplementary Figure 3). The presence of aggregates and degradation products indicates instability of the scFv antibody molecule, possibly explaining the low protein yield obtained from this scFv-hERG1 preparation.

### Production of a mutagenized scFv-hERG1

Since the instability and low protein yield of the “native” scFv-hERG1 could hinder further scaled up production and *in vivo* applications, we looked for strategies to improve the stability and yield of the antibody. From *in silico* analysis, we noticed the presence of a Phenylalanine (Phe) residue in position 92 of the VH sequence, between Framework 3 and CDR3, as resulted from the Kabat numbering scheme. This position often exhibits a conserved Cysteine (Cys) residue, crucial for the formation of a disulfide bond. Therefore, we decided to change the Phe into a Cys residue (see the arrow in Figure 1A), through site-directed mutagenesis. The T nucleotide in position 284, within the TTT codon, was substituted with a G, hence generating a TGT codon. The mutagenized construct, which carries the proper 284T>G mutation (Supplementary Figure 4), and therefore a Cys in position 92, was used to transform GS115 P. pastoris host yeast strain. We then chose the best (D8) scFv-hERG1-Cys expressing clone (Supplementary Figure 5). SDS-PAGE and Coomassie Brilliant Blue staining analysis of larger-scale cultures showed neat and evident bands in Fr. 12–14 (Figure 1C). A single, net and high peak, at the proper elution time was evident in the SEC chromatogram (Figure 1E). Overall the scFv-hERG1-Cys antibody shows good purity and stability. Consistently, the total amount of scFv-hERG1-Cys obtained from one liter of yeast culture was 1 mg, significantly higher compared to that obtained from the “native” scFv-hERG1.

### Evaluation of antigen affinity of the “native” and mutagenized scFv-hERG1

**Figure 2:**
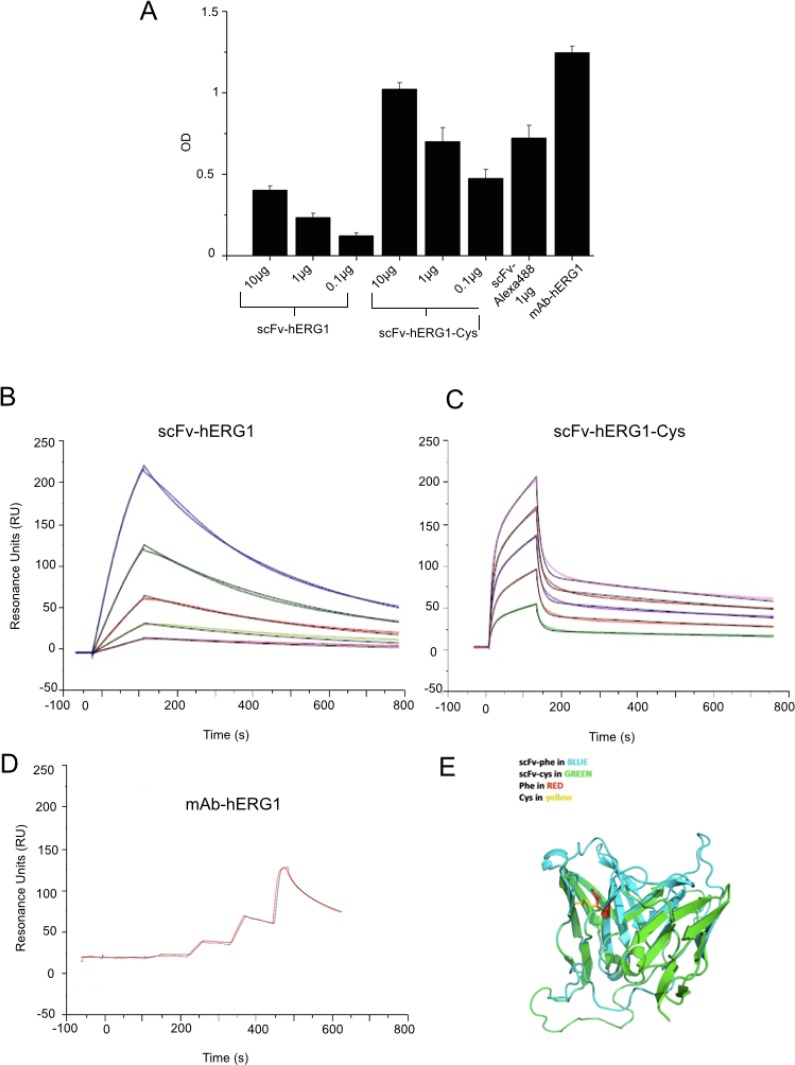
Antigen affinity and 3D structure modeling of scFv-hERG1 and scFv-hERG1-Cys antibodies (**A**) ELISA assay performed using the S5-Pore peptide (sequence: EQPHMDSRIGWLHN) as coating antigen and testing different amounts (from 0.1 to 10 μg) of scFv-hERG1 and scFv-hERG1-Cys antibodies. The scFv-hERG1-Cys-Alexa488 antibody was tested at 1 μg. As a control, 1 μg of the full length mAb-hERG1 antibody was used. In case the ELISA assay is performed using mAb-hERG1, the procedure differs, as the 2 h incubation with the monoclonal antibody is followed by the revealing using secondary peroxidate anti-mouse antibody (no intermediate incubation with anti-tag antibodies, e.g. anti-His, occurs). Values are expressed as OD_450_ and are means ± SEM of two independent experiments. Sensograms showing the results from SPR (Surface Plasmon Resonance) analysis at different concentrations of the scFv-hERG1 (**B**) and the scFv-hERG1-Cys (**C**) and hERG1-mAb (**D**) antibodies: violet 2.5 μg/mL, green 5 μg/mL, red 10 μg/mL, grey 20 μg/mL, and blue 40 μg/mL. (**E**) Modeled three dimension structure of the scFv-hERG1 (indicated as scFv-Phe in blue) and scFv-hERG1-Cys (indicated as scFv-Cys in green) through the SwissModel Expasy modeling tool. The Phe and Cys residues involved in disulfide bond formation are indicated in red and yellow, respectively.

The immunoreactivity of both “native” scFv-hERG1 and mutagenized scFv-hERG1-Cys was first tested through a sandwich ELISA assay, using the immobilized S5-Pore peptide as antigen. Figure 2A shows the results, expressed as Optical Density (O.D.). Both antibodies recognized the antigen in a dose-dependent manner, with a roughly two-fold higher binding of the scFv-hERG1-Cys compared to the “native” scFv-hERG1. Figure 2A also shows the O.D. values obtained with 1 μg of the mAb-hERG1 from which the scFv antibody was generated, as well as with an Alexa-488 labelled scFv-hERG1-Cys, that was used for further experiments, and is commented below.

Overall, the mutagenized scFv-hERG1-Cys displayed higher binding capacity towards the antigenic peptide, compared to the “native” scFv-hERG1. We deepened the binding properties and affinity of the two scFv antibodies by Surface Plasmon Resonance (SPR). The intact mAb-hERG1 was analyzed for comparison. The binding responses of antibodies, flowed over the immobilized S5-Pore peptide at different concentrations, were recorded in sensorgrams (Figure 2B–2D) and experimental values were fitted to kinetic models (Table 1). High quality fittings, as indicated by a residual χ^2^ values < 2%, were obtained for both scFv antibodies, although with different kinetics. The best fitting for the “native” scFv-hERG1 was obtained by applying the “1:1 binding model”, whereas for the scFv-hERG1-Cys the “two-state reaction model” gave the best result. This suggests that the two scFv antibodies have different modes of interaction with the antigen (see Discussion). The K_D_ value of the scFv-hERG1-Cys turned out to be lower compared to the “native” scFv-hERG1 (62 nM vs 318 nM, respectively) and closer to that of the intact mAb-hERG1 (16 nM) (Table 1). This value is the result of faster dissociation (lower Koff) and slower association (higher Kon) rates. This is also evident from the trend of the curves. Moreover, the Resonance Unit (RU) turned out to be 1021 for scFv-hERG1-Cys and 501.1 for the “native” scFv-hERG1. Overall the scFv-hERG1-Cys shows a much higher affinity for the antigen compared to the “native” scFv-hERG1. Finally, we used the SWISS-MODEL homology-modeling server to generate the 3D structure of the two scFv fragments. Both scFv antibodies showed correct core folding with very little conformational differences in the region carrying the mutation. Careful analysis of electrostatic surface potential and hydrophobicity did not indicate large differences between the two scFvs, other than in the loop and linker regions (Figure 2E and Supplementary Figure 6).

**Table 1:** Parameters derived from SPR analysis for the scFv-hERG1, scFv-hERG1-Cys and mAb-hERG1

Concentration	Kon (1/Ms)	Koff (1/s)	Ka2 (1/s)	Kd2 (1/s)	KD (M)	R max (Ru)	Chi2 (Ru2)	*U*-value
scFv-hERG1	2.06E^+04^	6.55E^–04^			3.18E^–07^	501.1	7.96	1
scFv-hERG1-Cys	1.46E^+05^	8.07E^–02^	6.40E^–03^	8.07E^–04^	6.18E^–08^		4.1	N/A
mAb-hERG1	1.595E^+6^	2.584E^–02^			1.621E^–8^	109.3	1.85	10.00

Values deriving from the SPR analysis related to Kon (1/Ms), Koff (1/s), Ka_2_ (1/s), KD (M), R max (RU), Chi^2^ (Ru^2^), *U*-value. The SPR angle change is reported as resonance units (RU), where 1000 RU correspond to an angle change of ∼ 0.1°. If the added molecule does not bind to a target or receptor, the SPR angle change in the sample and reference flow cells will be the same, and, after subtraction, will give a zero net RU response that indicates no binding occurred.

In conclusion, the mutagenized scFv-hERG1-Cys displayed higher stability, purity and affinity towards the raising peptide, compared to the “native” scFv-hERG1, and was hence chosen for further characterizations, both *in vitro* and *in vivo*.

### Characterization of scFv-hERG1-Cys immunoreactivity

**Figure 3:**
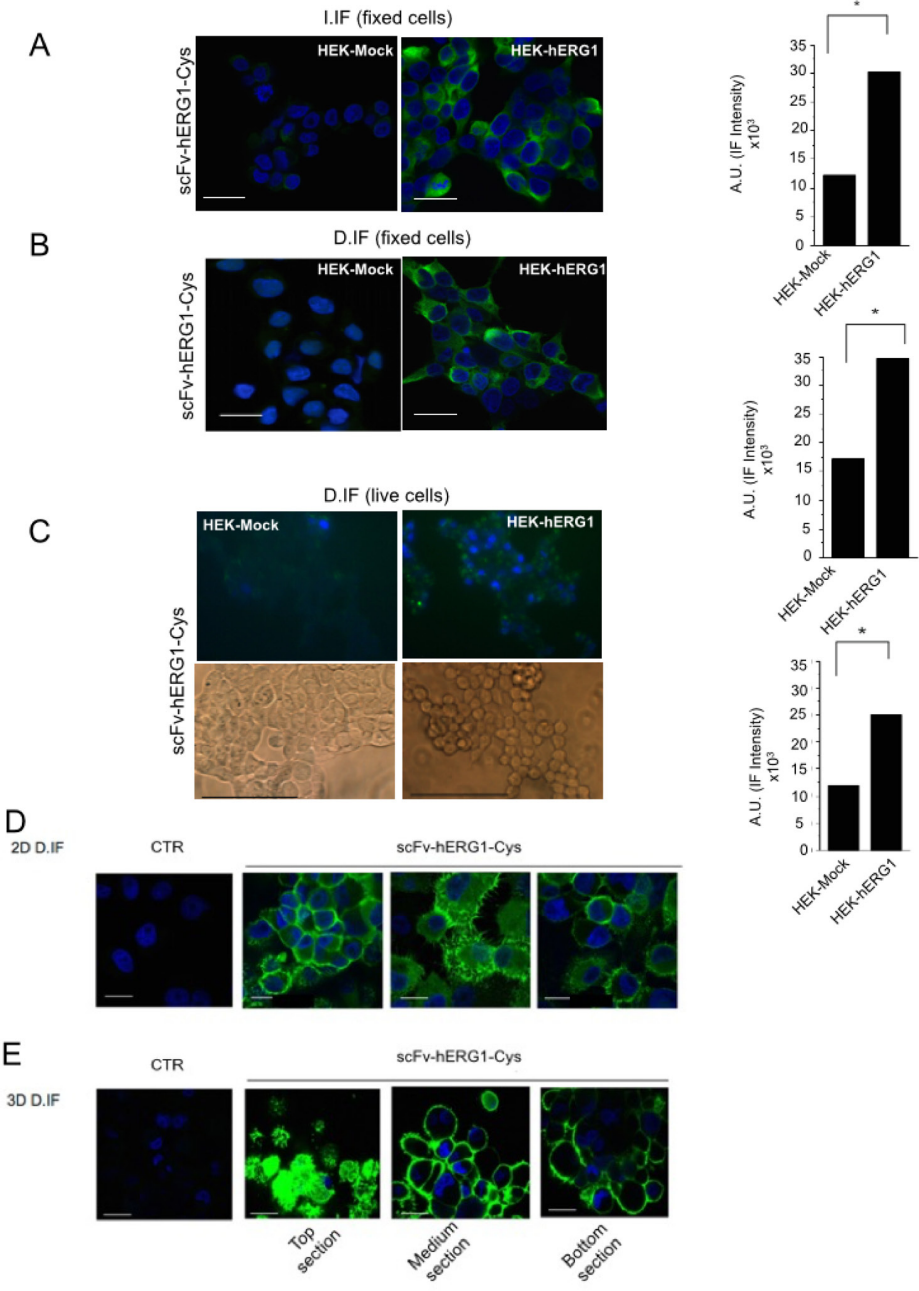
Immunofluorescence with scFv-hERG1-Cys (**A**) Indirect immunofluorescence (I-IF) on fixed HEK-Mock and HEK-hERG1 cells. Representative of 3 independent experiments performed in each cell line; the corresponding densitometric results are given in the bar graph on the right. Each image is representative of eight total images per experimental condition, analyzing ten cells per each. *P* values were calculated in respect to HEK-Mock cells using the Student’s *t* test, *p* < 0,05. (**B**) Direct immunofluorescence (D-IF) on fixed HEK-Mock and HEK-hERG1 cells using the scFv-hERG1-Cys-Alexa488. Representative of three independent experiments performed in each cell line; the corresponding densitometric results are given in the bar graph on the right. and the values obtained from the densitometric analyses of eight different images, considering ten cells each, *P* < 0,05 values were calculated in respect to HEK-Mock cells using the Student’s *t* test. (**C**) Direct immunofluorescence (D-IF) on live HEK-Mock and HEK-hERG1 cells using the scFv-hERG1-Cys-Alexa488. Representative of three independent experiments performed in each cell line; the corresponding densitometric results are given in the bar graph. Brightfield images are reported in the insets. *P* values were calculated in respect to HEK-Mock cells using the Student’s *t* test, *p* < 0,05. Bar = 100 µm. (**D**) Direct immunofluorescence (D-IF) on fixed PANC-1 cells cultured in 2D using the scFv-hERG1-Cys-Alexa488. The picture labelled as “CTR” represents cells incubated with only the secondary antibody. Representative of three independent experiments. Bar = 100 μm. (**E**) Direct immunofluorescence (D-IF) on fixed PANC-1 cells cultured in 3D as spheroids, using the scFv-hERG1-Cys-Alexa488. TOP, MEDIUM and BOTTOM sections of the spheroids are shown. The picture labelled as “CTR” represents cells incubated with only the secondary antibody. Representative of 3 independent experiments. Bar = 200 μm (first and third field); Bar = 100 μm (second field).

The immunoreactivity of the scFv-hERG1-Cys was first tested, through immunofluorescence (IF), towards the native hERG1 protein expressed on the plasma membrane of either normal or cancer cells. Both hERG1- and Mock- (as negative controls) transfected HEK 293 cells (HEK-hERG1 and HEK-Mock, respectively) were used to this purpose. We performed both indirect IF (I. IF) on fixed cells and direct IF (D. IF), on either fixed or live cells, employing an Alexa 488-conjugated scFv-hERG1-Cys. Representative pictures are in Figure 3A–3C, along with densitometric analyses. The scFv-hERG1-Cys antibody specifically recognized the native antigen, being the signal significantly higher in HEK-hERG1 cells compared to HEK-Mock cells in all the experimental conditions. The good signals obtained with D-IF indicate that the antibody maintained the capacity of recognizing the antigen even after direct conjugation with the fluorophore, confirming the ELISA assay in Figure 2A.

We then tested the immunoreactivity of the Alexa 488-labeled scFv-hERG1-Cys on cancer cells, which express the hERG1 protein at high levels [9]. Figure 3D shows data obtained with the Pancreatic Ductal Adenocarcinoma (PDAC) cell line PANC-1: a clear signal is evident at the plasma membrane level, with no evident diffused fluorescence due to unspecific Alexa 488 binding. The Alexa 488-conjugated scFv-hERG1-Cys was also tested in PANC-1 cells cultured in 3D as spheroids [22]. We detected a high and clear signal, in all the images taken at different Z planes (top, medium and bottom) (Figure 3E), indicating a good penetration of the antibody into the cellular mass, better than the intact mAb-hERG1 (Supplementary Figure 7).

### Characterization of scFv-hERG1-Cys: effects on cell viability

**Figure 4:**
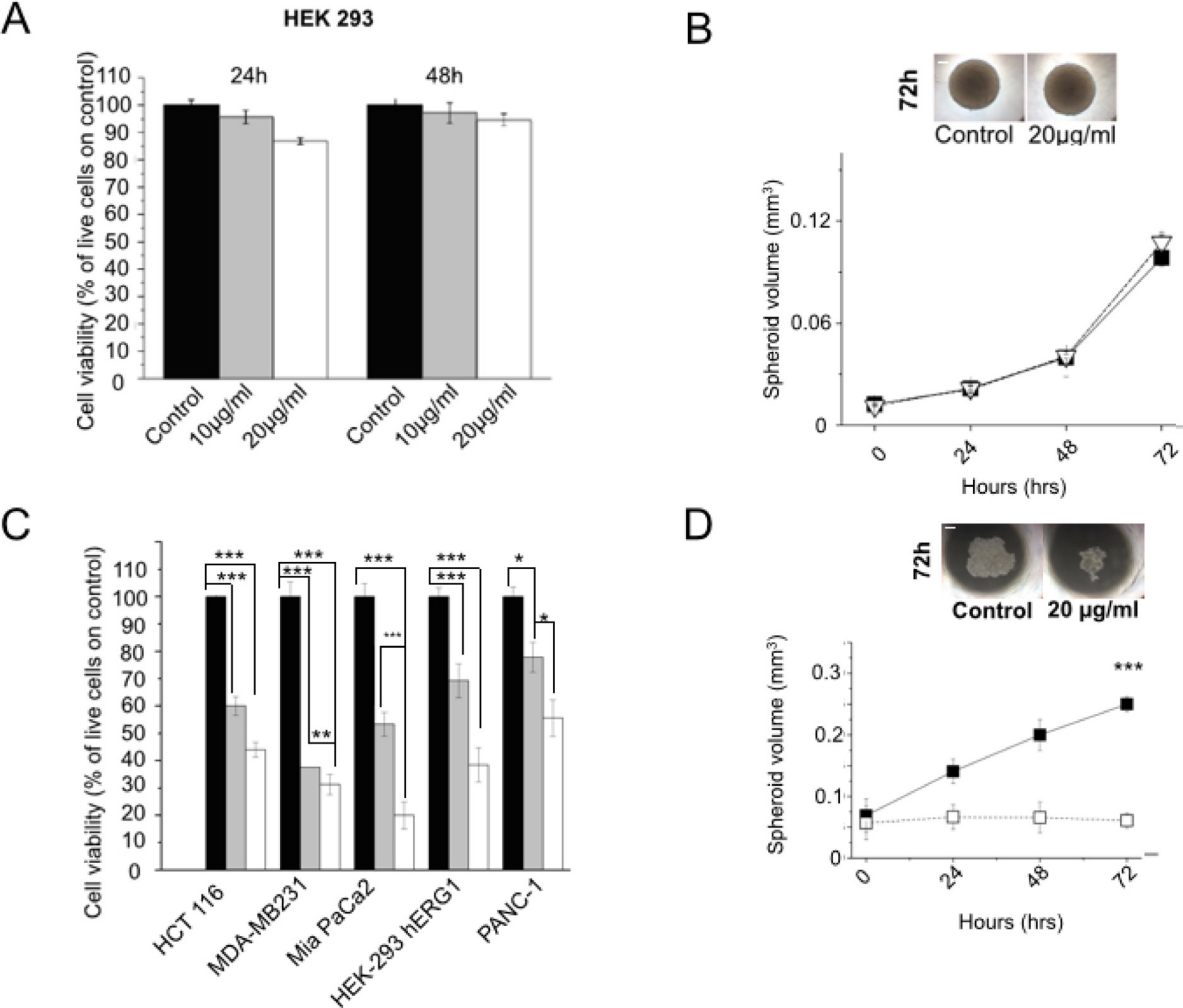
Effects of scFv-hERG1-Cys antibody on cell vitality of normal and cancer cells (**A**) Effects of the scFv-hERG1-Cys on cell viability of HEK 293 cells. Cell viability was assessed by the Trypan blue exclusion test after 24 h and 48 h of treatment with 10 and 20 μg/ml of the antibody. Values are expressed as percentage of viable cells and are means ± SEM of four independent experiments. (**B**) Effect of the scFv-hERG1-Cys (20 μg/ml) on the growth of HEK 293 cells cultured as spheroids. Black symbols: control; white symbols: scFv-treated spheroids. Spheroids in control conditions and after 72 h treatment with scFv-hERG1-Cys are shown in the inset on the top. (**C**) Effects of scFv-hERG1-Cys on cell viability of HEK 293-hERG1 cells and different cancer cell lines (MDA-MB231, MiaPaCa-2 and PANC-1). Cell viability was assessed by the Trypan blue exclusion test after 24 h of treatment with 10 (grey bars) and 20 (white bars) μg/ml of the antibody. Values are expressed as percentage of viable cells and are means ± SEM of three independent experiments. (**D**) Effect of the scFv-hERG1-Cys (20 μg/ml) on growth of Mia-Paca 2 cells cultured in 3D as spheroids. Black symbols: controls; white symbols: scFv-treated spheroids. Spheroids in control conditions and after 72 h treatment with scFv-hERG1-Cys are shown in the inset on the top. Statistical analysis was performed applying Bonferroni test: *p* values < 0,05 were considered significative (^**^); *p* values < 0,01 were considered highly significative (^***^).

We then analyzed the effects of the scFv-hERG1-Cys on cell viability: the antibody, used at either 10 or 20 μg/ml, had no effects on the viability of wild type HEK 293 cells, that do not express hERG1, neither after 24 or 48 hours (Figure 4A). Furthermore, the higher dose of the scFv (20 μg/ml) did not affect the size and morphology of HEK 293 spheroids up to 72 hours (Figure 4B). On the contrary, scFv-hERG1-Cys added for 24 hours to HEK 293 hERG1 cells, which over-express hERG1, or to different cancer cell lines (triple negative breast cancer cell line, MDA-MB231, pancreatic ductal adenocarcinoma cell lines, PANC-1 and MIAPaCa-2, colorectal cancer cell line, HCT-116) endowed with high endogenous hERG1 expression, significantly decreased cell viability (Figure 4C). Consistently, longer incubations (up to 72 hours) significantly reduced spheroid volume of both HEK-hERG1 (Supplementary Figure 8) and cancer cells. Data relative to MIAPaCa2 are in Figure 4D.

Overall, while the scFv-hERG1-Cys has no effects on vitality of normal cells, it efficiently binds and exerts functional activities (decrease of cell vitality and growth rate) on hERG1 expressing tumor cells.

### Serum stability and pharmacokinetics of the scFv-hERG1-Cys

**Figure 5:**
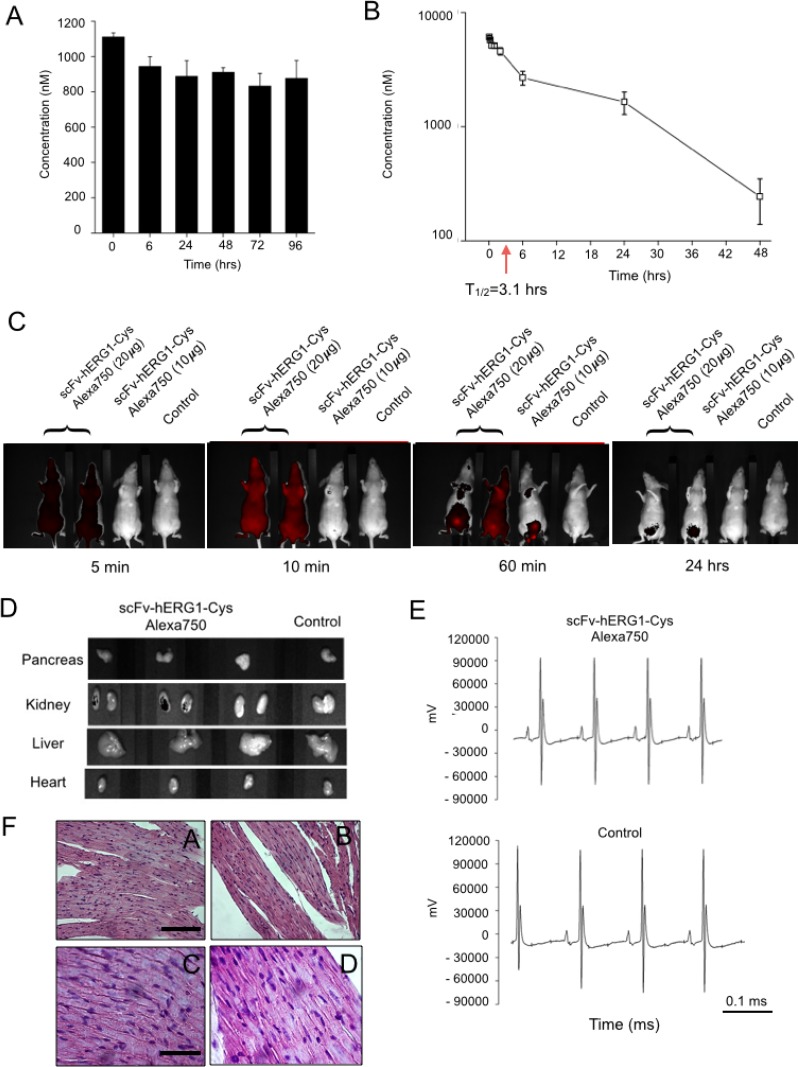
*In vivo* characterization of scFv-hERG1-Cys antibody in healthy mice (**A**) Serum stability of scFv-hERG1-Cys. Antibody concentration was determined by sandwich ELISA at different time points and is expressed as nanomolar concentration of the antibody. Values are means ± SD of two separate experiments. (**B**) Pharmacokinetics of scFv-hERG1-Cys. Immunodeficient nu/nu mice were injected i.v. with 160 μg of the antibody. Plasma was collected by tail vein puncture 5, 15, 30 min and 1, 2, 6, 24 and 48 h after scFv injection and the antibody concentration was determined by sandwich ELISA. Values are means ± SD of data collected in two different injected mice. (**C**) *In vivo* imaging of Alexa 750 labelled scFv-hERG1-Cys in healthy mice. Each panel shows the fluorescent signal in a mouse treated with scFv-hERG1Cys antibody conjugated with Alexa750 compared to a control mouse treated with PBS solution. Two mice were injected i.v. with 20 μg of antibodies, while one animal was injected with 10 μg. The fluorescent signal of the labeled scFv, was detected at different time points (5, 30, 60 minutes and 24 hours) from the administration. (**D**) Representative pictures of fluorescence analysis on dissected organs have been reported (pancreas, liver, kidneys and heart) collected after 24 hours from the injection of the scFv antibody. No fluorescent signal was detected, except for the kidneys, which showed a very weak fluorescent signal. Autofluorescence was subtracted based on the signal relative to WT non-injected mouse (Control). (**E**) ECG of a Control mice (upper panel) and a mouse treated with 20 μg of scFv (lower panel). The non-torsadogenic effect of the scDb was evalued considering no alterations were found either in the ECG trace and on ECG (Control mouse: QT interval = 34 ms, bpm = 380, QTc = 86. Treated mouse: QT interval = 36 ms; bpm = 372, QTc = 90 ms). No significant cardiac alterations were present. (**F**) Representative images of H&E (Hematoxylin and eosin) staining performed on heart sections deriving from immunodeficient nude mice injected with scFv-hERG1-Cys-Alexa750 antibody showing no evident signs of citotoxicity. Left panels, Control. Right panels. Section derived from mice injected with scFv-hERG1-Cys-Alexa750. Scale bars, left panels, 100 μm. Scale bars, right panels, 50 μm.

The serum stability of the scFv-hERG1-Cys was then assessed to determine whether it is stable against proteolytic activities. The antibody was incubated at 37° C in mouse serum up to 96 hours and its concentration was determined through a sandwich ELISA assay. After 96 hours, roughly 80% of the original antibody was present (Figure 5A), demonstrating that the scFv-hERG1-Cys is relatively stable in serum.

The pharmacokinetic (PK) properties of scFv-hERG1-Cys were studied after intravenous (i.v.) injection into immunodeficient nude mice, i.e. the same strain used for testing the Alexa-labelled scFv antibody in optical imaging (see below). The protein was injected at 160 μg/mouse and the plasma concentration was determined at different time points by a sandwich ELISA assay. The scFv-hERG1-Cys showed a characteristic two-phase pharmacokinetic behavior, with a rapid distribution phase and a longer elimination phase (Figure 5B). The half-life of the elimination phase turned out to be 3.1 hours.

### *In vivo* optical imaging

The suitability of the scFv-hERG1-Cys for imaging applications was then analyzed. To this purpose, 10 and 20 μg of an Alexa 750 conjugated scFv-hERG1-Cys were injected i.v. into immunodeficient nude mice, and the distribution of the labelled scFv was determined. To this purpose, we collected NIR images at different time points during the distribution phase, i.e. between 5 and 60 minutes (see also Figure 5B). A clear and broad fluorescent signal was visible 5 and 10 minutes after injection, with high ROI (region of interest) values (Table 2), which started to decay at 60 minutes. A low signal remained evident in the lower abdominal area, very likely corresponding to kidney and bladder, 24 hours after antibody injection, in mice treated with the higher (20 μg) antibody dose. At the same time point, no signal was evident in the mouse injected with the lower (10 μg) antibody dose (Figure 5C). At 24 hours the main organs were explanted and NIR signals evaluated: no NIR signal could be detected in explanted organs, except a scanty signal in the kidneys (Figure 5D). Neither signs of suffering were observed in the injected mice, during their monitoring for 10 days after inoculum, nor evident organ toxicity were detected at the sacrifice (Supplementary Figure 9). In particular, and most importantly, the scFv-hERG1-Cys had no gross effects on ECG parameters, in particular on QT interval values (Figure 5E and related legend), as well as on the morphology or cardiac myocytes (Figure 5F).

**Table 2:** ROI values obtained in healthy mice (*n* = 3) after i.v. of scFv-hERG1-CysAlexa750 at 5, 10, 60 min, 24 h. Values are normalized on controls

	scFv-hERG1-Cys Alexa750 (1) cpm/cm^2^ 20 µg	scFv-hERG1-Cys Alexa750 (2) cpm/cm^2^ 20 µg	scFv-hERG1-Cys Alexa750 (3) cpm/cm^2^ 10 µg
5 min	766.6	766.6	115
10 min	683.3	683.3	158.3
60 min	316.6	550	233.3
24 h	51.6	53.3	25

**Figure 6:**
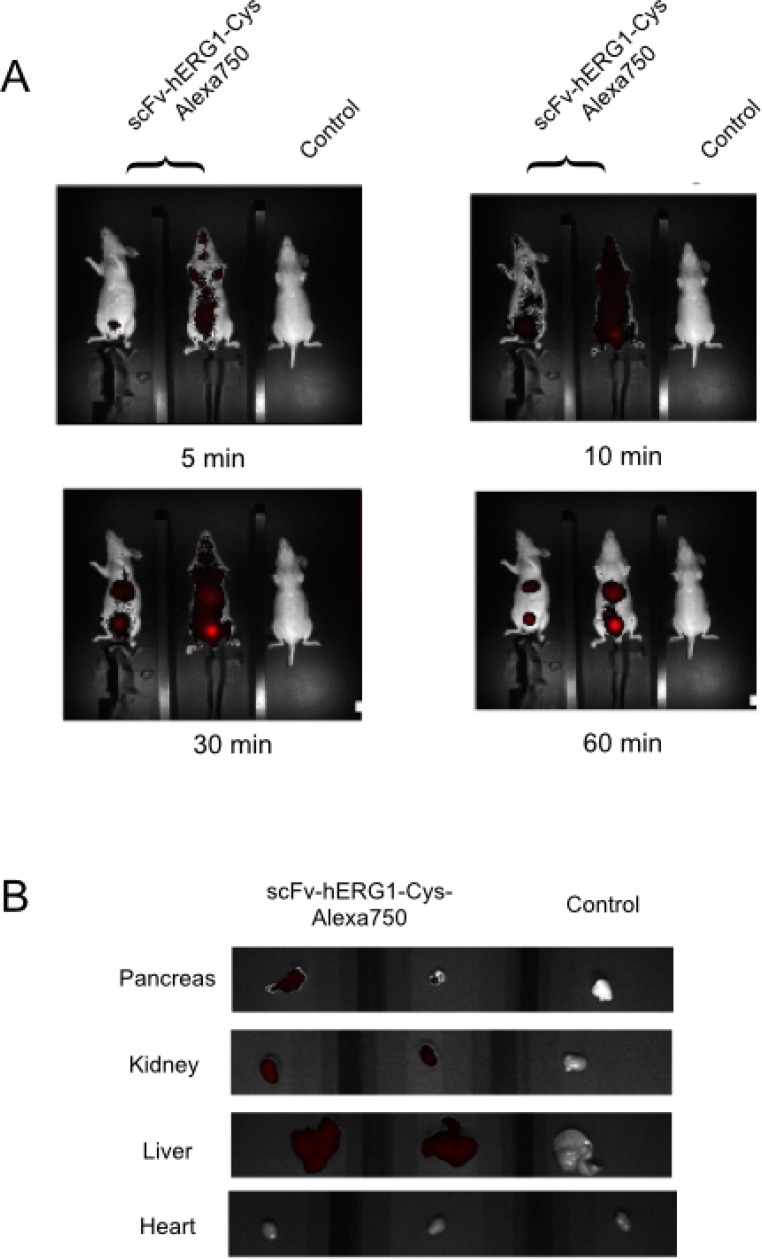
*In vivo* distribution of the scFv-hERG1-Cys-Alexa750 antibody in a pancreatic tumor bearing mice (**A**) scFv-hERG1-D8Cys-Alexa750 uptake and retention of scFv-hERG1-D8Cys-Alexa750 in a MIAPaCa-2-nu/nu mice model of PDA. Mice were administered through tail vein injection with 6.5 μg of scFv-hERG1-D8Cys-Alexa750 antibody. Representative pictures of mice i.v. injected with the labelled antibody (left) have been compared with control mice (right). Fluorescence intensity in the abdominal area, the site proximal to tumor has been analyzed. ROI values are reported in Table 3A. (**B**) Representative pictures of fluorescence analysis on dissected organs have been reported. Pancreas showed a clear fluorescent signal for the organ treated with the scFv-Alexa750 antibody while hearts did not show fluorescent signal in either the antibody treated mouse, as well as in the control mouse. Autofluorescence was subtracted based on the signal relative to WT non-injected mouse (Control).

Hence, we decided to use the higher (20 μg/mouse) dose of the Alexa-labelled scFv-hERG1-Cys antibody for testing its ability to bind hERG1 in a cancer tissue *in vivo*. To this purpose, we used an orthotopic PDAC mouse model (i.e. immunodeficient nude mice injected with MIAPaCa-2 cells in the pancreas as in [20]). Forty-five days after tumor-cell injection, when a big tumor mass inside the pancreas and many metastatic foci were present in the liver ([20] and Supplementary Figure 10), the Alexa 750 conjugated scFv-hERG1-Cys was injected i.v. and NIR images collected. A significant fluorescent signal was already detected 5 min after antibody injection, reaching an overall maximum level between 30 and 60 minutes (Figure 6A). The signal was evident especially in the upper abdominal area, with ROI values significantly higher than those obtained, in the same area, in healthy mice (Table 3A). On the contrary, the signal in the lower abdominal area was roughly similar in PDAC and healthy mice. Sixty minutes after antibody injection, mice were sacrificed, the principal organs (liver, heart and pancreas) were collected and ex vivo NIR imaging measurements were performed. A clear fluorescent signal was detected in the tumor masses within the pancreas and in the metastases within the liver of mice treated with the scFv-hERG1-Cys-Alexa750 antibody, compared to control mice. Lower signals were detected in the kidney, the main protein excretory organ, in injected mice, whereas no signal could be detected in the heart of both injected and not injected mice (Figure 6B). ROI values allowed to calculate the tumor-to-organ ratio of the labelled scFv antibody (Table 3B).

**Table 3A:** ROI values obtained in tumor-bearing mice (*n* = 2) after i.v. of scFv-hERG1-CysAlexa750 at 5, 10, 30, 60 min

	scFv-hERG1-Cys Alexa750 (1) cpm/cm^2^	scFv-hERG1-Cys Alexa750 (2) cpm/cm^2^
5 min	13.3	5.7
10 min	333	233.3
30 min	581.6	250
60 min	500	310

**Table 3B:** ROI values obtained from tumor-bearing mice organs analysis *ex-vivo*

	scFv-hERG1-Cys Alexa750 (1) cpm/cm^2^	scFv-hERG1-Cys Alexa750 (2) cpm/cm^2^	Tumor to organ ratio
Pancreas	60.3	1.9	105.5
Liver	17.6	30	25.3
Hear	24.3	27	1

Values are normalized on controls.

Overall, the Alexa750-conjugated scFv-hERG1-Cys antibody is capable of reaching its target (i.e. the hERG1 protein overexpressed in PDAC cells) and is retained in the tumor masses for enough time to allow its detection by NIR imaging techniques.

## DISCUSSION

Molecular imaging can be used for the detection and staging of primary tumors, as well as for post treatment follow up of cancer patients. The major limitation of molecular imaging is the lack of specificity of most of the probes commonly applied. Hence, the development of imaging agents with higher binding capacity and selectivity to cancer is of great importance. Antibody-based molecular imaging would be most suitable for this purpose, provided that tools with retained affinity, improved pharmacokinetics and high tumor-to-organ contrast ratios are developed. Smaller molecules, such as antibodies in the single chain format, scFv, are optimal candidates to fit such requirements [1–5]. In the present study we generated a single chain antibody (scFv) against a novel cancer biomarker (i.e. the hERG1 potassium channel) and provide a preliminary proof of concept that it has the appropriate features (easy production protocols, good stability and immunoreactivity, favourable pharmacokinetic and tumor-to-organ ratios *in vivo*) to be exploited for *in vivo* cancer diagnostics in the near future.

The generation of high affinity antibodies from libraries is often difficult [22], hence we used a hybridoma cell line secreting relevant monoclonal antibodies (mAb) as the starting point for recombinant antibody work. The scFv we generated was in fact derived from the cloned V_H_ and V_L_ regions of an anti hERG1-mAb we produced in the past [18]. This “native” scFv-hERG1 suffered from a very low yield, that we traced back to the formation of aggregates as a result of instability of the antibody. The deduced amino acid sequence of the cloned scFv showed a Phe residue in position 92 of the V_H_ domain, a site that usually contains a conserved Cys residue, usually involved in the formation of a disulfide bond between the b- and f- β strands of the immunoglobulin domain, with the corresponding Cys in position 22. Such disulfide bond is quite conserved in antibodies and does not appear necessary for proper function of antibodies while it confers stability to the molecule [23]. Only a few natural antibodies have been described, in which the disulfide bridge in the V_H_ domain is lacking, because of the missing of either the 22 or 92 Cys residues [24]. In particular, the anti-ABPC48 antibody, although lacking the Cys in position 92 [25], maintains it functionality beacuse the Cys residue is replaced by a strong hydrophobic residue, Tyrosine [26], which preserves the hydrophobic core [27] and hence antibody performances. Similarly, in our “native” scFv antibody, a hydrophobic aminoacid replaces the Cys residue in position 92. This substitution likely occurs also in the natural hERG1-mAb (see the sequences of the pool of cloned V_H_s in Supplementary Figure 11. Consistent with what occurs in the anti-ABPC48 antibody, the natural hERG1-mAb shows good performances and affinity constants (low K_D_: 16 nM), while the scFv derived from it (the “native” scFv-hERG1) is unstable and shows a decreased affinity (higher K_D_, see Table 1). This is an expected finding, due to the univalency of the scFv [28]. On the contrary, the mutagenesis procedure we applied was capable to generate a single chain antibody, the scFv-hERG1-Cys, with advantageous affinity constants, closer to those of the bivalent monoclonal antibody (K_D_ 62 nM). In addition, the mutagenized scFv was more stable, without aggregates or degraded forms (Figure 1E), and hence with higher manufacturing performances. Furthermore, as derived from the sensorgrams in Figure 2B and 2C and the related fitting models, the two scFv antibodies display different kinetics, although both with high quality fitting, as indicated by low χ^2^ values. The different kinetics suggests that the two scFv antibodies have different modes of interaction with the antigen. In particular, the scFv-hERG1-Cys displayed a more complex kinetics, fitted through the “two-state reaction model”, which suggests a possible structural rearrangement of the “antibody-antigen” complex. However, a deeper understanding of such possible conformational changes which might occur after the scFv binds to its antigen is outside the scope of this study. For our purposes, the finding of K_D_ values more favorable for the scFv-hERG1-Cys represents the cornerstone for further antibody characterizations. Indeed, the mutagenized scFv-hERG1-Cys also showed good performances when tested in living systems. First of all, it showed a good labelling signal in hERG1-expressing cells, either hERG1-transfected HEK cells or cancer cells, which endogenously express the channel. In the latter cells, the engineered antibody was able to decrease cell proliferation (in 2D cultures) and the growth of 3D spheroids, further indicating its specificity for the hERG1 biomarker. On the contrary, it did not show any effect on the viability of normal cells in which hERG1 is not expressed, when cultured *in vitro*. More importantly, the scFv-hERG1-Cys did not show any acute or chronic toxicity when injected in healthy mice. In particular, we did not detect neither functional (ECG) or morphological (histology) cardiac alterations, and a very low accumulation of the 750 Alexa-labelled scFv-hERG1-Cys in the heart. Although rodents are not the optimal models wherein to analyze potential side effects of hERG1 targeting, our results are encouraging proof of concepts to further proceed to regulatory analyses. Such good performances *in vivo* were also accompanied by a good stability in serum at 37° C, indicating resistance to protease activity, and by a rapid half-life, 3.1 hours, nor far from what commonly reported for scFv antibodies [29, 30]. Another good feature of our scFv was its good penetration into 3D spheroids, witnessed by a good labeling at any level of the cellular mass (Figure 3E), accompanied by functional activities (reduction of spheroid growth) at concentrations comparable to those commonly used for these types of experiments [12]. The latter feature stresses the capability of the short antibody molecule to penetrate into cellular masses, a relevant prerequisite Overall, the above characteristics are relevant prerequisites for the applicability of the scFv-hERG1-Cys as an imaging tool to detect tumor masses *in vivo*.

Furthermore, encouraging performances were shown by the Alexa-labelled scFv-hERG1-Cys antibodies. First of all, the labeling with an Alexa fluorophore did not alter the affinity (Figure 2A) and immunoreactivity of the antibody on cells (either fixed or live) (Figure 3). Good data also emerged from the Alexa 750-labelled antibody tested *in vivo* (Figures 5 and 6). In fact, the fluorescent antibody rapidly distributed in healthy mice, showed a fast clearance and did not accumulate into organs, including the heart (see the comments above). Furthermore, when used in an orthotopic model of PDAC cancer, we found a good accumulation into the abdominal area, corresponding to sites (pancreas and liver) with the presence of tumor masses. This was also confirmed by *ex vivo* data. What is more, the single chain antibody showed a very good tumor-to-tissue ratio since it was shown to accumulate within the neoplastic pancreas and the metastatic liver and no relevant fluorescent signal was detected in the heart. The tumor-to-organ ratios we obtained are compatible with what reported with other scFvs used for *in vivo* imaging applications [31–33] and better compared to whole antibody molecules [34].

Overall, our data indicate that the scFv-hERG1-Cys could be a good candidate as a diagnostic tool, with fast and less expensive production and purification protocols, suitable for *in vivo* imaging in those cancers in which hERG1 overexpression and its prognostic relevance has already been shown.

## MATERIALS AND METHODS

### Cloning of the heavy and light chains of the hERG1 mAb

The heavy and light chain cDNA of the hERG1-mAb was PCR-amplified after reverse transcription of the total RNA extracted from A7 hybridoma using SuperScript^®^ II Reverse Transcriptase, (Thermo Fisher, Massachusetts, USA), according to manufacturer’s instructions.

According to Wang and colleagues [21], the V_H_ region was amplified using five different degenerated forward primers that anneal to the framework 1 (FR1) of the variable domain (degH1 to degH5) and a reverse primer that anneals to the constant region of the immunoglobulin isotype of the hERG1 mAb, i.e. IgG2b. The V_L_ region was amplified using a degenerated forward primer and a canonical reverse primer, both relative to the κ type light chain, degL(κ)_forward and κ_reverse, respectively. The cDNA was amplified using Phusion^®^ High-Fidelity DNA Polymerase (Finnzymes Reagents). The agarose gel showing V_H_ and V_L_ amplification is shown in Supplementary Figure 12 and the primers’ sequences are reported in Supplementary Table 1 in the Supplementary Materials.

After electrophoresis gel separation and DNA purification, the two V_H_ and V_L_ PCR fragments, were independently cloned into pCR-Blunt (Invitrogen) vector following manufacturer’s instructions, and the resulting plasmids were used to transform DH5 α E. coli cells.

Colonies were screened to verify the presence of the insert, both by excising the insert through digestion with NotI (New England BioLabs, Massachusetts, USA) and by PCR amplification (using the primers reported in the Supplementary Table 1) of the extracted plasmid DNA. The extracted DNAs from eight colonies were sequenced through Automated DNA sequencing service (PRIMM) and the most common sequence was chosen. Some representative sequences obtained in pCR-Blunt vector are reported in Supplementary Figure 11.

### Cloning into pHenIX expression vector

V_H_ and V_L_ sequences were then cloned into the pHenIX phagemid, which contains the peptide linker sequence necessary to join the carboxy terminus of V_H_ to the amino terminus of V_L_, thus allowing the proper assembly of the scFv antibody. First, appropriate restriction sites were added to the V_H_ and V_L_ sequences by PCR, using the primers reported in Supplementary Table 2.

After cloning into pHenIX phagemid, the proper orientation of the insert was verified through Sanger sequencing resulting in only one colony containing the V_H_-linker-V_L_ sequence properly inserted and oriented (see Supplementary Figure 13).

### Cloning of scFv in pPIC9K expression vector

To clone the scFv-hERG1 into the pPIC9K vector, suitable for protein expression in P. pastoris yeast cells, the scFv expression cassette in the pHenIX vector was amplified by PCR using primers introducing the FspI and AvrII restriction sites at 5′ and 3′ ends of the sequence, respectively (reported in Supplementary Table 3). Restriction sites were inserted using Phusion^®^ High-Fidelity DNA Polymerase (Finnzymes Reagents), with the following PCR cycling conditions: 94° C for 2 min, 28 cycles of a three-step program (98° C for 30 sec; 59° C for 30 sec and 72° C for 30 sec) followed by a final extension step of 72° C for 10 minutes. After separation of the PCR product through agarose gel electrophoresis, the band of the appropriate molecular weight (870 bp) was excised from the gel and purified using QIAquick PCR Purification Kit (QIAGEN, Hilden, Germany). The DNA was then cut with FspI and AvrII to allow its ligation into the pPIC9K vector that had been cut with Eco53KI and AvrII restriction enzymes (New England BioLabs, Massachusetts, USA).

### scFv-hERG1 mutagenesis

Mutagenesis was performed on the scFv-hERG1 expression cassette cloned into pPIC9K using the QuikChange^®^ XL Site-Directed Mutagenesis Kit (Stratagene, Agilent Technologies, California, USA), according to the manufacturer’s instructions, using the following primers (Primm Biotech): forward primer: GGATTCTGCAGTCTATTACTGTGCAACAGGTTGGGGACCTG; reverse primer: CAGGTCCCCAACCTGTTGCACAGTAATAGACTGCAGAATCC. DH5α competent cells were transformed with the DNA obtained after the mutagenesis protocol and plasmid DNA extracted from different colonies was sequenced to verify the presence of the desired mutation.

### scFv expression in Pichia pastoris

The scFv construct in pPIC9K was linearized with SalI and used to transform the P. pastoris strain GS115 using the spheroplasting technique. The Pichia Expression Kit (Thermo Fisher, Massachusetts, USA) protocol was followed.

### Purification using ÄKTA chromatography systems

The purification of yeast supernatants (1 liter) obtained from scaled up cultures, was performed by Affinity Chromatography, using an ÄKTA Protein Purification System (Ge Healthcare Life Sciences, Illinois, USA) using a HisTrap HP 1 ml column (Ge Healthcare Life Sciences, Illinois, USA). Wash steps and equilibration were performed according to the manufacturer’s instructions, using Wash buffer (20 mM sodium phosphate, 500 mM NaCl, pH 7.3). Elution was performed through a linear gradient of Elution buffer (20 mM sodium phosphate, 500 mM NaCl, 500 mM imidazole, pH 7.3). Chromatogram analysis was accomplished using UNICORN 7.0 software.

### Gel Filtration

Samples obtained from purification were dialyzed against 20 mM sodium phosphate, 150 mM NaCl, pH 7.3 buffer using Slide-A-Lyzer™ Dialysis Cassettes (Thermo Fisher, Massachusetts, USA) and gel filtered, using Superdex 75 HR 10/30 (Ge Healthcare Life Sciences, Illinois, USA). According to the characteristics of the column (Superdex 75, Ge Healthcare Life Sciences, Illinois, USA), proteins with a molecular weight of around 30 KDa, like scFv, should have a retention time of roughly 24–25 minutes. Wash buffer composition was adjusted to optimize protocol conditions (20 mM sodium phosphate, 150 mM NaCl, pH 7.3). Eluted solutions were analyzed through SDS-PAGE.

### Sodium dodecyl sulphate polyacrylamide gel electrophoresis (SDS-PAGE) and western blotting

SDS and Western Blot analysis were performed according to [16].

### Sandwich ELISA assay

Sandwich ELISA was performed following standard method, using anti-6xHis antibody (Abcam, Cambridge, UK) 1:250 in PBS + 3%BSA to reveal the scFv, followed by anti-mouse IgG-HRP conjugate (Sigma, Missouri, USA) 1:500 in PBS + 3%BSA, as described in [35]. In case the ELISA assay is performed using mAb-hERG1, the procedure differs as the 2 h incubation with the monoclonal antibody is followed by the revealing using secondary peroxidate anti-mouse antibody (no intermediate incubation with anti-tag antibodies, e.g. anti-His, occurs).

### Pharmacokinetic analysis

2 Balb/c mice have been injected with 160 μg of scFv-hERG1-Cys antibody and blood samples have been collected from the tail vein at 0, 5, 15, 30, 120, 360, 1440, 2880 minutes after antibody injection. Each sample was spun at 12000 rpm for 5 minutes; the resulting plasma was stored at −80° C until analyzed. The plasma concentration of scFv-hERG1-Cys antibody was determined by sandwich ELISA, using anti-6xHis antibody (Abcam, Cambridge, UK) 1:250 in PBS + 3%BSA to reveal the scFv, followed by anti-mouse IgG-HRP conjugate (Sigma, Missouri, USA) 1:500 in PBS + 3%BSA. The half-lives for elimination phase were determined using Origin 7.0 Software by fitting the last four data points into the first-order equation, T_1/2_ = (∆t/t_1_-t_0_)/∆C where (∆t/t_1_-t_0_) represents the slope of the curve and ∆C represents the value corresponding to the half of the antibody concentration at t_1_.

### Surface plasmon resonance analysis

The affinities of the scFv and mAb-hERG1 antibodies were measured by the surface plasmon resonance (SPR) method, using a Biacore T100 and a Biacore T200 instrument, respectively (GE Healthcare, Illinois, USA). For the scFv analyses, the S5-Pore peptide was covalently immobilized to the flow cell of a carboxymethylated dextran CM5 sensor chip using an amine-coupling strategy. Successful immobilization was performed with 7 injections of 540 s each, 5 µl/min, in 10 mM acetate buffer at pH 4.5. The two scFv were then flowed over immobilized S5-Pore peptide at different concentrations (2.5, 5, 10, 20, and 40 μg/ml), according to the following conditions: contact time, 120 sec; dissociation time, 600 sec; flow, 30 µl/min. Regeneration was performed using 100 mM Glycine, pH 2.5. MAb-hERG1 was bound to a Protein G pre-coated CM5 sensor chip to reach a level of immobilization of 7693 relative units (RU). S5-Pore was then applied at different concentrations (10 nM, 50 nM, 100 nM, 500 nM) using the Single Cycle kinetics method to derive binding kinetics, according to the following conditions: contact time 30 s, dissociation time 120 s, flow 30 µl/min. Regeneration was performed using 100 mM Glycine, pH 1.7. Data analysis was performed using BIA Evaluation software v 3.1.

### Cell culture

HEK-hERG1 cells, HEK-MOCK and PANC-1 cells were routinely cultured in bidimensional (2D), as described in detail in the Supplementary Materials. Mia Paca2 cells were cultured as in [20]. 3D cultures (spheroids) were performed following the protocol described in [22].

### Immunofluorescence (IF)

HEK-hERG1 cells, HEK-MOCK and PANC-1 cells were routinely cultured in bidimensional (2D). Both direct and indirect IF were performed as in [15, 21]. Details are reported in the Supplementary Materials.

### Cell viability assay

Cell viability was evaluated performing a Trypan blue assay. Briefly, cells were seeded in a 96-well plate at a density of 5 × 10^3^ cells/well. The following day, the medium was replaced with 100 µl of fresh medium containing different concentrations of the scFv-hERG1-Cys antibody (10 μg/ml and 20 μg/ml). After 24 hours and 48 hours incubation, cells were detached and viable cells were counted. All the experiments were carried out in triplicate.

### Antibody structure modelling

Antibody modeling was performed through the SWISS-MODEL (ExPASy) protein structure homology-modelling server [36, 37]. We have not referred to a target model alignment. In fact, if the target-template sequence identity is lower than 40%, the alignment generally has gaps and needs manual intervention to minimize the number of misaligned residues

### Antibody labelling with Alexa 488 and Alexa 750 fluorophore

The scFv-hERG1Cys was conjugated with Alexa Fluor^®^ 488 Microscale Protein Labeling Kit (Thermo Fisher Scientific, Massachusetts, USA), according to the indications in the protocol. We have calculated the DOL (Degree of Labelling) according manifacturer’s instructions and it turned out to be 2.2.

Conjugation with Alexa 750 (Thermo Fisher Scientific, Massachusetts, USA) was performed after adjusting the labelling protocol, as no commercial kits suitable for the labelling are available. To individuate the best labelling conditions, three different quantities of dye were used to react with 150 μg of scFv-hERG1-Cys: condition 1–4 µl of Alexa Fluor^®^ 750 NHS Ester; condition 2–8 µl of Alexa Fluor^®^ 750 NHS Ester; condition 3–12 µl of Alexa Fluor^®^ 750 NHS Ester. The scFv-hERG1-Cys at a concentration of 2 mg/ml in PBS solution and 0.1 M sodium bicarbonate buffer pH 8.3, was incubated 1 hour at 22° C in agitation with the different amount of Alexa Fluor^®^ 750 NHS Ester (Succinimidyl Ester) (Thermo Fisher Scientific, Massachusetts, USA), resuspended in DMSO at 10 mg/ml. The reaction was blocked for 5 minutes in ice and the labelled protein was purified by size exclusion chromatography on a Sephadex G25 (Sigma, Missouri, USA) column equilibrated with PBS. The concentration of protein in the three conditions was initially calculated by the equation:

$$\text{protein concentation}{=\text{A}}_{\text{protein}}\times \text{protein m.w.}{\text{/Σ}}_{\text{protein}}$$

where A_protein_= A_280_–A_max_× 0,04, with A_max_ (absorbance of dye at ≤_max_) determined at 749 nm and 0,04 = contribution of the dye to the absorbance at A_280_ (CF Alexa750 = 0,04).

Subsequently the degree of labelling (D.O.L., corresponding on the number of dye molecules present on the labelled protein) was calculated for the three conditions by the equation:

$$\text{D.O.L.}=\left({\text{A}}_{\text{max}}\text{×protein m.w.}\right)\text{/}\left(\left[\text{proteina}\right]{\text{×Σ}}_{\text{dye}}\right)$$

where ∑_dye_ = 290000 cm^–1^ M^–1^

D.O.L. for each condition resulted to be 1.48 (condition 1), 2.17 (condition 2) and 4.25 (condition 3). One aliquot of proteins from each labelling condition was controlled on 15% SDS PAGE (Supplementary Figure 14) confirming the data obtained by D.O.L. calculation: for condition 1 is evident an increase of molecular weight of protein of 1300 g/mol (dye molecular weight), for condition 2 an increase 2600 g/mol and and for condition 3 and increase of 5200 g/mol. Since the condition 2 approximately presents the same D.O.L. obtained by labelling with Alexa Fluor^®^ 488 Microscale Protein Labeling Kit, it was chosen for *in vivo* experiments.

### *In vivo* analysis

*In vivo* imaging: All experiments involving mice were approved by the Italian Ministry of Health. *In vivo* experiments were performed by the LIGEMA, a joint laboratory of University of Florence and Dival Toscana srl, at the Animal Facility of the University of Florence. Three six-week old, female immunodeficient Athymic Nude-Foxn1 nude mice were injected intravenously with 50 µl (1 nm dye/mouse) of scFv-hERG1-Cys labeled with the fluorophore Alexa 750 and fluorescence was measured 5, 10, 60 minutes and 24 hours after antibody injection. One control mouse was treated with sterile PBS solution. All the fluorescent emission spectra were measured using a Photon imager (Biospace Lab). The imager had a laser source for fluorescence excitation (λ = 679 nm), an emission filter (λ = 702 nm) for fluorescence detection, and a computer for data analysis.

The fluorescent signal of the fluorophore was detected with the optical imager PhotonImager (BiospaceLab); the set-up of the acquisitions was: FLI integration (shutter: 1000 ms, 4 s per frame) Fluorophore: Alexa 750, Excitation = 749 nm, Emission = 775 nm, Lens: diaphragm = 1.4 focus = 87.0% Aperture autopilot: yes Stage height: 440 mm Software version: 2.11.0. All the collected scans were elaborated with the software M3vision, in order to save the picture and quantificate the signal detected (in terms of ROI).

ECG measurements were performed using VevoLAZR-X imaging station and calculated with Vevo LAB Analysis Software. To analyze the mice ECG we used Vevo LAB software, measuring the time (ms) between the start of the Q wave and the end of the T wave, in order to assess the QT interval. The QTc interval was calculated using the Bazett formula. Subsequently, the ECG data (mV and ms) were extracted and the ECG curve was reproduced by using Origin software.

Mouse Model: the MIAPaCa-2 cell line was used for tumor cell implantation, as described in [20]. Cells were cultured in DMEM supplemented with L-glutamine (4 mM), 10% fetal bovine serum and Geneticin (G418) (2.4 mg/ml) (Gibco, Massachusetts, USA) at 37° C in a humidified atmosphere of 5% CO_2_. MIAPaCa-2-luc cells were injected into the pancreas of nude mice and the animals were monitored (as described in [20]) and 45 days after the cell inoculum, mice were administered with scFv-hERG1Cys-Alexa750 antibody.

### Statistical analysis

Statistical analysis was performed applying Shapiro-Wilk test and Bartlett test to assess data normality and homoscedasticity assumptions respectively. Associations between continuous variables and categorical variables were analyzed through Anova; subsequently, to determine pairwise statistical significance Tukey test (^*^p < 0,05) or Bonferroni test have been applied. *p* values < 0,05 were considered significative; *p* values < 0,01 were considered highly significative.

## SUPPLEMENTARY MATERIALS FIGURES AND TABLES



## Abbreviations

ANOVA: Analysis of variance
BSA: Bovine serum albumin
Cys: cysteine
CDR: Complementarity-determining region
CTR: Control
D. IF: Direct Immunofluorescence
ECG: ElectroCardioGram
ELISA: Enzyme-Linked ImmunoSorbent Assay
Fv: Fragment variable
I. IF: Indirect Immunofluorescence
I.F: Immunofluorescence
HRP: Horse radish peroxidase
mAb: monoclonal antibody
NSH: N-Hydroxysuccinimide
O.D: Optical Density
Phe: phenilalanine
PK: Pharmacokinetic
RU: Response units
PDAC: Pancreatic Ductal Adenocarcinoma
PCR: Polymerase chain reaction
ROI: Region of Interest
scFv: Single chain fragment variable
SEC: Size-exclusion chromatography
SPR: Surface Plasmon Resonance
TRAIL: tumor necrosis factor-related apoptosis: inducing ligand
V_H_: Variable heavy
V_L_: Variable light
