# Supplementary material for: Generation and characterization of novel recombinant anti-hERG1 scFv antibodies for cancer molecular imaging
Source: Oncotarget. 2018 Oct 9;9(79):34972–89. doi: 10.18632/oncotarget.26200 (PMC6201861; doi:10.18632/oncotarget.26200)
Supplement: Supplementary file 1 [file oncotarget-09-34972-s001.pdf]

Generation and characterization of novel recombinant anti-hERG1 scFv antibodies for cancer molecular imaging

SUPPLEMENTARY MATERIALS

MATERIALS AND METHODS

Screening of clones after transformation in P. Pastoris

After five days from transformation, single colonies were visible to the naked eye and were picked and transferred to 96-well plates. Selection was carried out exploiting the presence, in the pPIC9K vector, of the bacterial kanamycin gene that confers resistance to Geneticin®, treating the yeasts with two different concentrations of G418 (0.5 or 15 mg/ml). After two days of growth at 30° C, clones grown in 15 mg/ml G418 were picked up and evaluated for their capacity to express the protein of interest, setting up a small scale liquid culture, according to Pichia Expression Kit protocol (Invitrogen). Samples from each clone's culture were collected at different time-points: 24, 48, 72 hours. After three days of induction with 0.5% final concentration of methanol, supernatants were collected and tested through slot blot.

Slot blot analysis

Yeast supernatants (200 µl) were applied to a PVDF membrane (Amersham) assembled in a slot blot device between two sheets of 3 MM Whatman paper. The samples were left in incubation for 15 minutes. Blocking was performed with T-PBS 5% BSA for 45 minutes and the membrane was then washed 10 min with T-PBS. The membrane was finally incubated for 1 hour with anti-6xHis-HRP conjugated antibody (Sigma) diluted 1:2000 in T-PBS plus 5% BSA.

Ni Sepharose purification

Supernatants, obtained from small-scale yeast cultures, were incubated O/N in rolling with Ni Sepharose (6 Fast Flow Ge Healthcare Life Sciences). After two wash steps with 500 µl Wash Buffer (20 mM sodium phosphate, 500 mM NaCl, pH 7.3), elution was performed using 250 µl Elution buffer (20 mM sodium phosphate, 500 mM imidazole, pH 7.3).

Sodium dodecyl sulphate polyacrylamide gel electrophoresis (SDS-PAGE)

15 µl of each sample were separated on a 15% acrylamide gel. Electrophoretic runs were performed at 150 V. Gels were either stained with Coomassie Brilliant Blue or transferred to PVDF membranes (see below) for western blot analysis to assess the proper molecular weight (around 30 KDa) of the protein.

Western blot (WB)

After SDS-PAGE, gels were transferred to PVDF membrane (Amersham) in transfer buffer (200 mM glycine, 25 mM Tris, 20% methanol) at 100 V for 1 h. Membranes were washed in T-PBS (PBS 0.1% Tween) and then blocked O/N with T-PBS containing 5% BSA. Membranes were exposed to primary antibody peroxidase-coupled (Sigma) diluted in T-PBS containing 5% BSA for one hour at room temperature. After washing the membranes three times for 10 minutes, each signal was visualized using ECL reagent (Amersham). WB were performed using the following antibodies: anti-myc (Santa Cruz Biotechnology) 1:1000 and anti-6xHis-HRP conjugated antibody (Sigma) 1:2000.

Immunofluorescence protocol and analysis

Cells

HEK-hERG1 cells (HEK 293 cells stably transfected with the pcDNA3.1-hERG1 construct) and HEK-MOCK cells (HEK 293 cells stably transfected with pcDNA 3.1 empty vector) were grown overnight in DMEM medium with 10% FBS (Euroclone, Fetal Bovine Serum EU Approved, COD. ECS0180L) in a 37° C incubator with 5% CO_2, after seeding on glass coverslips (10^5 cells/ml).

Indirect IF (I-IF) on fixed cells

At the end of incubation, cells were washed once with PBS and fixed with 4% paraformaldehyde (PFA) for 20 min at room temperature. Blocking was performed with

10% BSA for 2 h at room temperature. I-IF was performed using scFv-hERG1-G3, scFv-hERG1-D8Cys, diluted to a final concentration of 20 µg/ml in blocking solution and incubated for 2.5 hours, followed by O/N incubation with the anti-His antibody (1:250) (Abcam) in a blocking solution. The following day, cells were washed three times with PBS and incubated with anti-mouse Alexa 488 (Invitrogen) for 1 hour. Cells were then incubated with Hoechst (1:1000) for 30 minutes and mounted with propyl gallate. Cells were visualized on a confocal microscope (Nikon, C1). IF quantification was performed using ImageJ software: for each image, the measure of three different areas was performed and the mean was calculated.

Direct IF (D-IF)

D-IF was performed using labelled scFv-hERG1 and scFv-hERG1-Cys. To this purpose, scFv-hERG1 and scFv-hERG1Cys were conjugated with Alexa Fluor® 488 Microscale Protein Labeling Kit (Thermo Fisher Scientific), according to the indications in the protocol. The scFvs were diluted 1:20 in blocking solution and incubated O/N at 4° C. IF on live cells was performed on cells grown O/N on 60 mm Petri dishes (Sarstedt) using an agarose (15 g/l) ring in order to isolate cells and minimize the volumes of reagents needed for incubations. The following day, cells were washed three times with PBS and incubated with Hoechst (1:1000) for 30 min and mounted with propyl gallate. Cells were visualized on a confocal microscope (Nikon, C1). IF quantification was performed as described for I-IF.

IF on spheroids

One thousand PANC-1 cells were seeded on an agarose base layer (1.5 g/l) in a 96 well plate and grown for 72 hours in a humidified incubator at 37° C and 5% CO_2. For IF experiments, spheroids were incubated with scFv-hERG1-D8Cys (conjugated with Alexa Fluor 488), following the protocol previously described for IF on live cells.

IF intensity (A.U.) calculated using Image J Software (ImageJ 1.38, U.S. National Institutes of Health). For each image, the mean of the fluorescence intensity of three different areas was calculated after the subtraction of the blue channels values (which refers to nuclei staining).

Primers used for the assembly of scFv-hERG1 antibody construct

Supplementary Table 1: List of primers used to amplify the V_H and V_L domains of hERG1 mAb antibody

	PRIMERS 5′-3′
degH2_for	GAGGTCCARCTGCAACARTC
IgG2b_rev	AGGGGCCAGTGGATAGACTGATGG
degL(κ)_for	GAYATTGTGMTSACMCARWCTMCA
κ_rev	GGATACAGTTGGTGCAGCATC

Supplementary Table 2: List of primers used to insert the proper restriction sites used to clone V_H and V_L domains in pHenIX vector

	PRIMERS 5′-3′
forward VL-*ApaL*I	acgcgtgcactgGATATTGTGCTGACACAATCT<br>CCA
VL-*Not*I	ataagaatgcggccgcGGATACAGTTGGTGCAGC<br>ATC
VH-*Sal*I,	acgcgtcgacGAGGTCCAACTGCAACAGTC
VH-*Xho*I	ccgctcgagccAGGGGCCAGTGGATAGACTGA<br>TGG

Supplementary Table 3: List of primers used to insert the proper restriction sites used to clone the scFv-hERG1 construct into pPIC9K vector

	PRIMERS 5′-3′
VH- *Fsp*I	AAAATGCGCAGAGGTCCAACTGCAACA<br>GTC
VL- *Avr*II	GGGGCCTAGGGGATACAGTTGGTGCAGC<br>ATC

GAGGTCCAACTGCAACAGTCTGGACCTGAACTGGTGAAGCCTGGGGCTTCTGTGAAGATATCCTGCAAGACTTCAGG
ATACACAT TCACTGAATACACCGTTCACTGGGTGAAACAGAGCCATGGAAAGAGCCTTGAATGG
ATTGGAGGCATTAATCCTAATGGTGGTACTACCTATAATCAGAAGTTCAAGGGCAA
GGCCACATTGACTATTGACAAGTCCTCCAGCTCAGCCTTCATGGAGCTCCGCAGCC
TGACATCTGAGGATTCTGCAGTCTATTACTTTGCAACAGGTTGGGGACCTGACTAC
TGGGGCCAAGGCACCACTCTCACAGTCTCCTCAGCCAAAACAACACCCCCATCAGT
CTATCCACTGGCCCCTGGCTCGAGTGGTGGAGGCGGTTCAGGCGGAGGTGGCTCTG
GCGGTAGTGCACTGGATATTGTGCTGACACAATCTCCACTCACTTTGTCGGTTAAC
ATTGGTCAACCAGCCTCTATCTCTTGCAAGTCAAGTCAGAGCCTCTTATATACTAA
TGGAAAAACCTATTTTAATTGGTTATTACAGAGGCCAGGCCAGTCTCCAAAGCGCC
TAATCTATCTGGTGTCTAAACTGGACTCTGGAGTCCCTGACAGGTTCACTGGCAGT
GGATCAGGAACAGATTTTACACTGAAAATCAGCAGAGTGGAGGCTGAAGATTTGGG
AGTTTATTACTGCGCGCAAGGTACACATTTTCCGTGGACGTTCGGTGGAGGGACCA
AGCTGGAAATCAAACGGGCTGATGCTGCACCAACTGTATCC

Supplementary Figure 1: scFv-hERG1 expression cassette sequence cloned in pPIC9K. Underlined are shown restriction sites; in red are primer sequences; in yellow is peptide linker sequence.

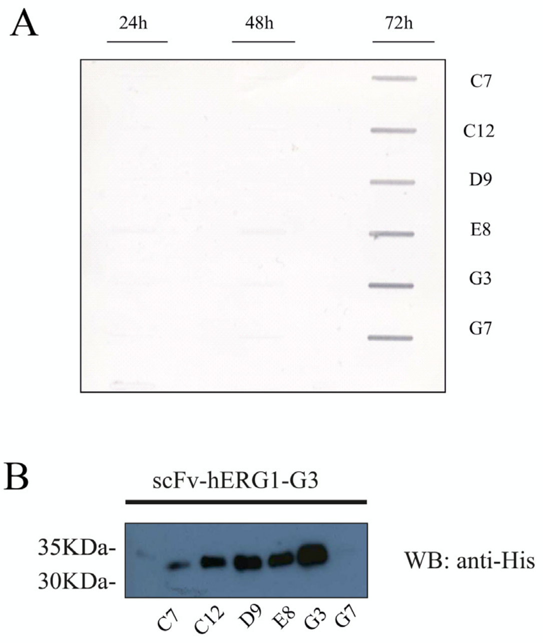

Supplementary Figure 2: (**A**) Slot blot on C7, C12, D9, E8, G3, G7 supernatants. (**B**) Western-blot analysis on C7, C12, D9, E8, G3, G7 purified clones of scFv-hERG1-G3.

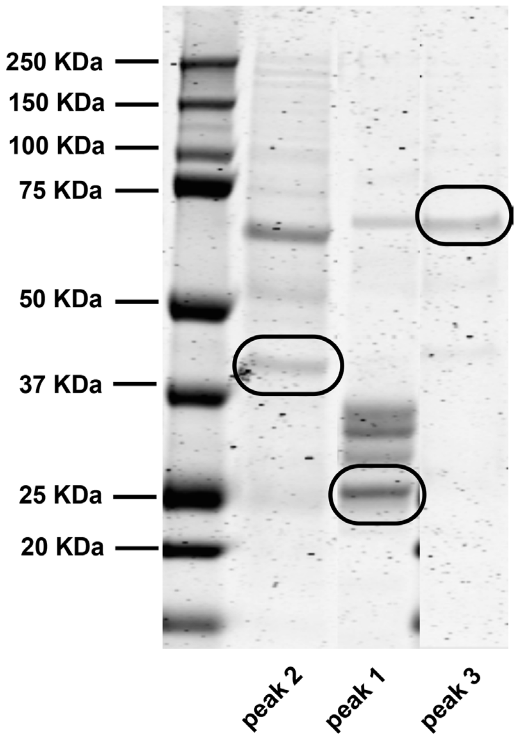

Supplementary Figure 3: Coomassie staining showing the analysis of peaks 1, 2, 3, obtained after SEC (Size exclusion chromatography).

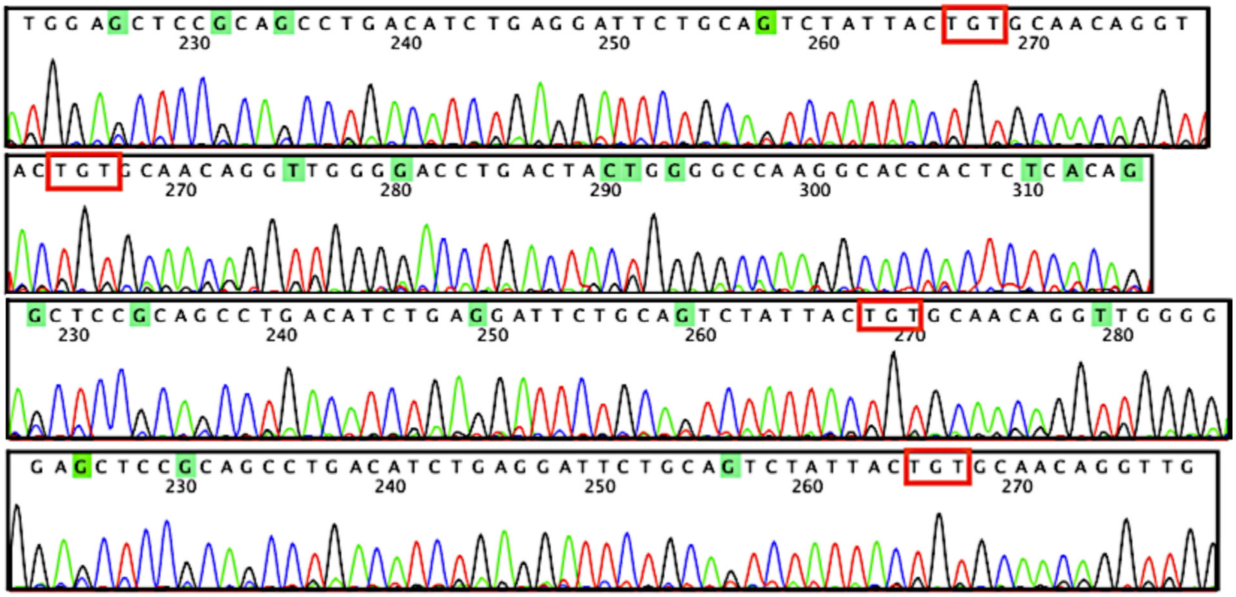

Supplementary Figure 4: Portions of DNA sequence obtained from four mutagenized scFv-hERG1 colonies, showing the proper mutation from TTT to TGT in position c284T>G.

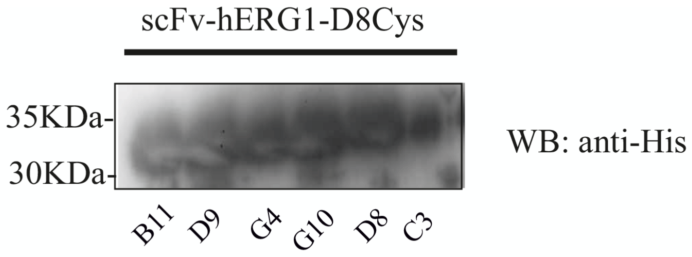

Supplementary Figure 5: Western-blot analysis on B11, D9, G4, G10, D8, C3 purified clones of scFv-hERG1-D8Cys.

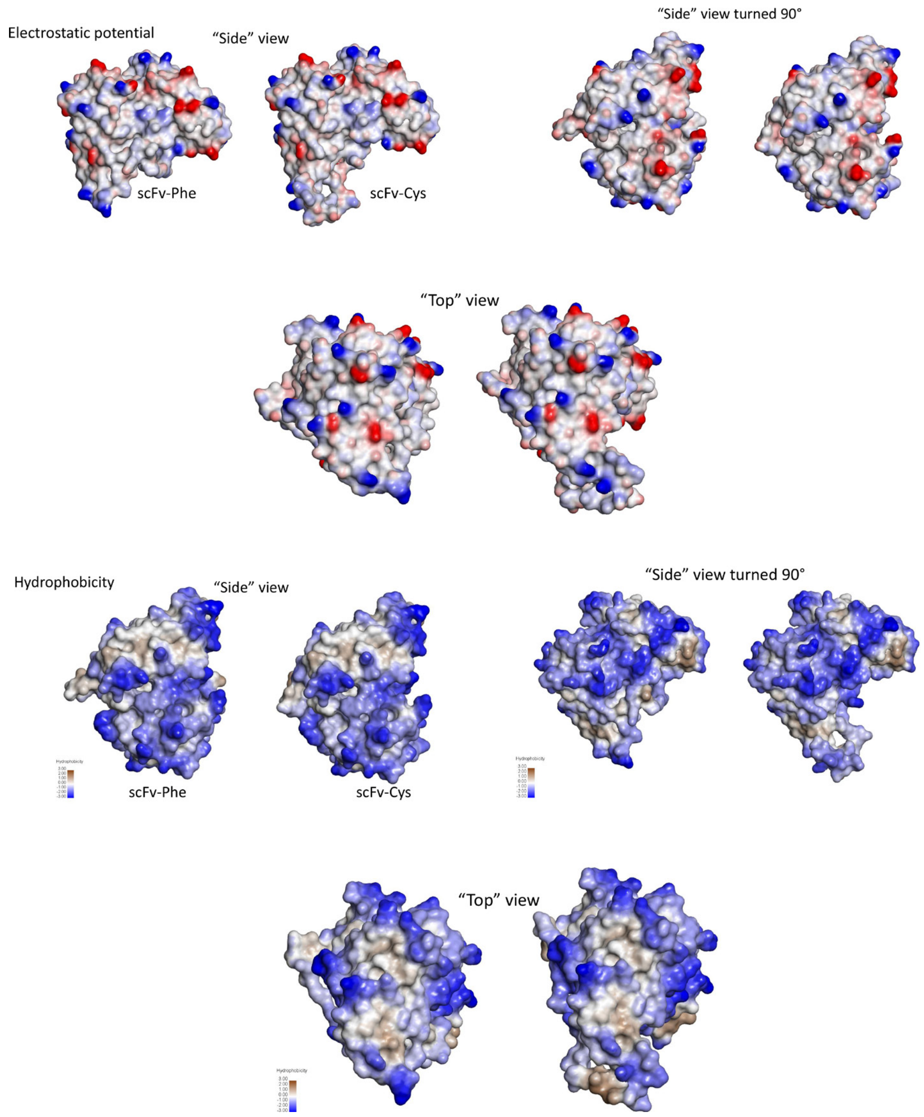

Supplementary Figure 6: 3D structure of scFv-hERG1 and scFv-hERG1-Cys antibodies showing both electrostatic and hydrophobicity characteristics. Both showed correct core folding with very little conformational difference in the region with the mutation. Careful analysis of electrostatic surface potential and hydrophobicty did not indicate large differences other than in the loop and linker regions. This is to be expected from such a modelling routine.

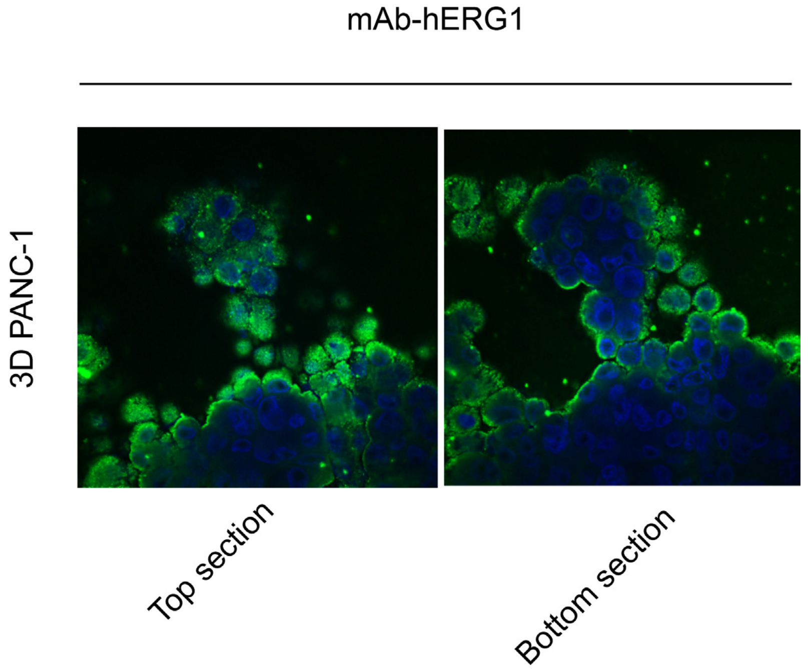

Supplementary Figure 7: IF showing results obtained on 3D PANC-1 spheroids stained with mAb-hERG1 antibody. We detected a membraneous signal at a lower extent for the intact mAb-hERG1 (20×10^3 AU for the mAb-hERG1 vs 35×10^3 AU for the bottom section Z-stack). The latter ImageJ analysis shows that the scFv antibody has better characteristics regarding the penetration into the cells.

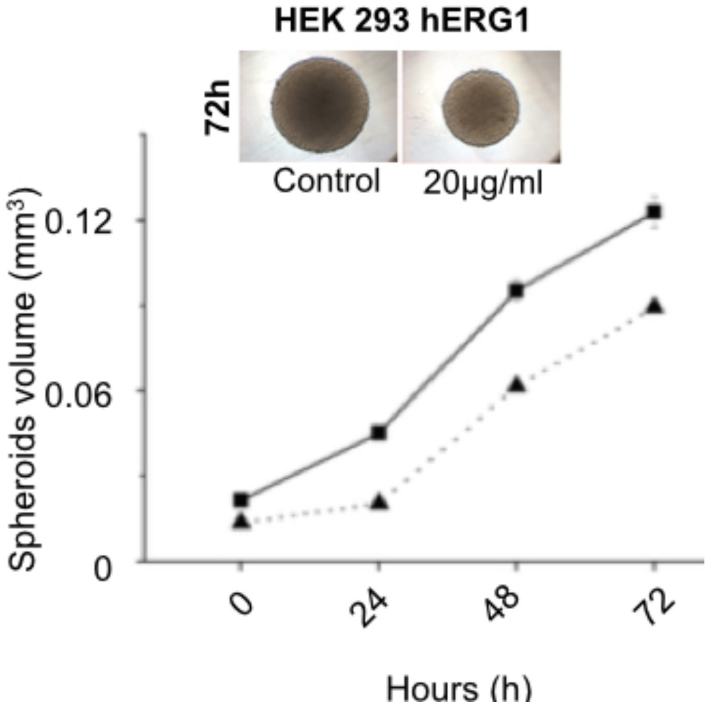

Supplementary Figure 8: HEK-hERG1 spheroid growth curve. Spheroids were treated with 20 μg/ml of scFv-hERG1-Cys antibody. Insets show spheroids representative images after 72 hrs treatment. scFv-hERG1-Cys has no effects on vitality of normal cells, while it efficiently binds and exerts functional activities (reduction of cell proliferation) on hERG1 expressing tumor cells.

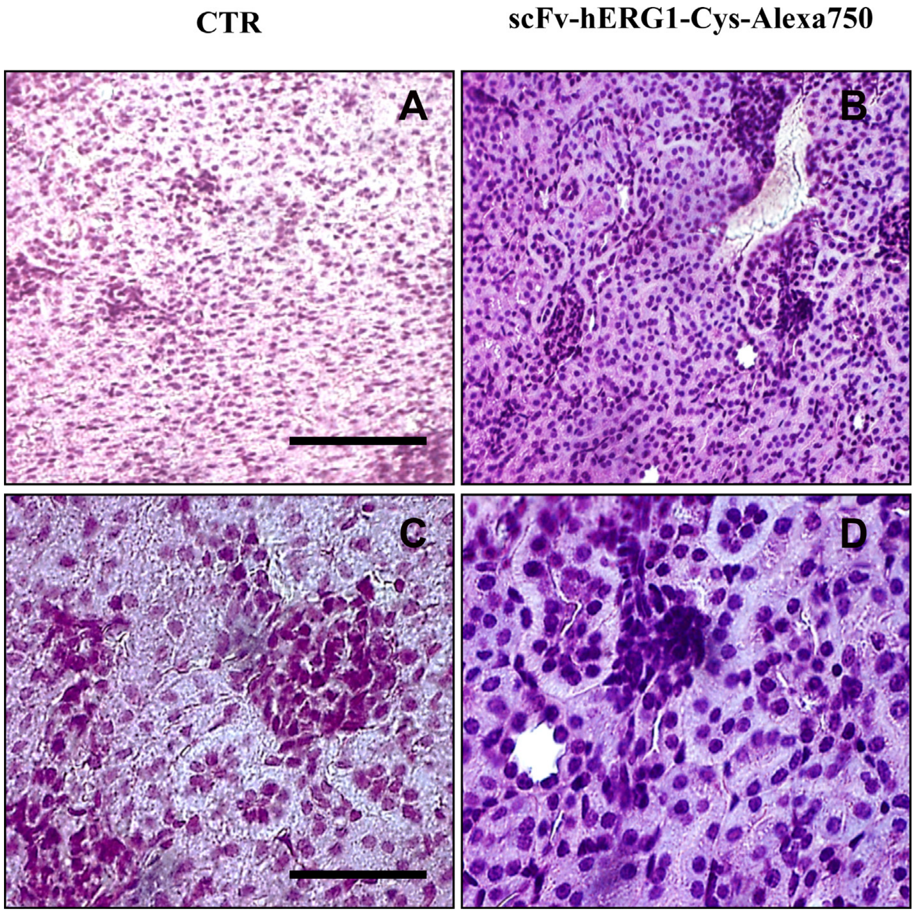

Supplementary Figure 9: Representative images of H&E (Hematoxylin and eosin) staining performed on kidney sections deriving from immunodeficient nude mice injected with scFv-hERG1-Cys-Alexa750 antibody showing no evident signs of citotoxicity. (Panel **A**, **C**) Control. (Panel **B**, **D)** Section derived from mice injected with scFv-hERG1-Cys-Alexa750. Scale bars, panels (**A**, **B**) 150 µm. Scale bars, panels (**C**, **D**) 100 µm.

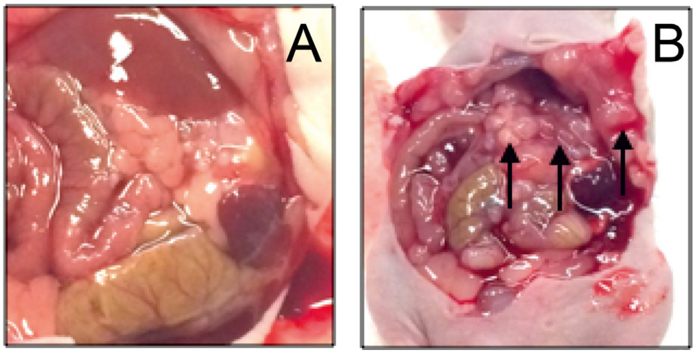

Supplementary Figure 10: Representative pictures of abdominal areas of tumor-bearing mice at the moment of sacrifice. As highlighted by the arrows many metastatic foci were visible.

mAb-hERG1 VH pCRBlunt-SEQU. N°1

GAGGTCCAACTGCAACAGTCTGGACCTGAACTGGTGAAGCCTGGGGCTTCTGTGAAGATATCCTGCAAGACTTCAGGAT
ACACATTCACTGAATACACCGTTCACTGGGTGAAACAGAGCCATGGAAAGAGCCTTGAATGGATTGGAGGCATTAATCC
TAATGGTGGTACTACCTATAATCAGAAGTTCAAGGGCAAGGCCACATTGACTATTGACAAGTCCTCCAGCTCAGCCTTCA
TGGAGCTCCGCAGCCTGACATCTGAGGATTCTGCAGTCTATTACTTTGCAACAGGTTGGGGACCTGACTACTGGGGCCA
AGGCACCACTCTCACAGTCTCCTCAGCCAAAACAACACCCCCATCAGTCTATCCACTGGCCCCT

mAb-hERG1 VH pCRBlunt-SEQU. N°2

GAGGTCCAACTGCAACAGTCTGGACCTGAACTGGTGAAGCCTGGGGCTTCTGTGAAGATATCCTGCAAGACTTCAGGATA
CACATTCACTGAATACACCGTTCACTGGGTGAAACAGAGCCATGGAAAGAGCCTTGAATGGATTGGAGGCATTAATCCTA
ATGGTGGTACTACCTATAATCAGAAGTTCAAGGGCAAGGCCACATTGACTATTGACAAGTCCTCCAGCTCAGCCTTCATGG
AGCTCCGCAGCCTGACATCTGAGGATTCTGCAGTCTATTACTTTGCAACAGGTTGGGGACCTGACTACTGGGGCCAAGGC
ACCACTCTCACAGTCTCCTCAGCCAAAACAACACCCCCATCAGTCTATCCACTGGCCCCT

mAb-hERG1 VH pCRBlunt-SEQU. N°3

GAGGTCCAACTGCAACAGTCTGGACCTGAACTGGTGAAGCCTGGGGCTTCTGTGAAGATATCCTGCAAGACTTCAGGATA
CACATTCACTGAATACACCGTTCACTGGGTGAAACAGAGCCATGGAAAGAGCCTTGAATGGATTGGAGGCATTAATCCTA
ATGGTGGTACTACCTATAATCAGAAGTTCAAGGGCAAGGCCACATTGACTATTGACAAGTCCTCCAGCTCAGCCTTCATGG
AGCTCCGCAGCCTGACATCTGAGGATTCTGCAGTCTATTACTTTGCAACAGGTTGGGGACCTGACTACTGGGGCCAAGGC
ACCACTCTCACAGTCTCCTCAGCCAAAACAACACCCCCATCAGTCTATCCACTGGCCCCT

mAb-hERG1 VH pCRBlunt-SEQU. N°4

GAGGTCCAGCTGCAACAGTCTGGACCTGAACTGGTGAAGCCTGGGGCTTCTGTGAAGATATCCTGCAAGACTTCAGGATA
CACATTCACTGAATACACCGTTCACTGGGTGAAACAGAGCCATGGAAAGAGCCTTGAATGGATTGGAGGCATTAATCCTAA
TGGTGGTACTACCTATAATCAGAAGTTCAAGGGCAAGGCCACATTGACTATTGACAAGTCCTCCAGCTCAGCCTTCATGGAG
CTCCGCAGCCTGACATCTGAGGATTCTGCAGTCTATTACTTTGCAACAGGTTGGGGACCTGACTACTGGGGCCAAGGCACC
ACTCTCACAGTCTCCTCAGCCAAAACAACACCCCCATCAGTCTATCCACTGGCCCCT

mAb-hERG1 VH pCRBlunt-SEQU. N°5

GAGGTCCAACTGCAACAATCTGGACCTGAACTGGTGAAGCCTGGGGCTTCTGTGAAGATATCCTGCAAGACTTCAGGATAC
ACATTCACTGAATACACCGTTCACTGGGTGAAACAGAGCCATGGAAAGAGCCTTGAATGGATTGGAGGCATTAATCCTAAT
GGTGGTACTACCTATAATCAGAAGTTCAAGGGCAAGGCCACATTGACTATTGACAAGTCCTCCAGCTCAGCCTTCATGGAG
CTCCGCAGCCTGACATCTGAGGATTCTGCAGTCTATTACTTTGCAACAGGTTGGGGACCTGACTACTGGGGCCAAGGCACC
ACTCTCACAGTCTCCTCAGCCAAAACAACACCCCCATCAGTCTATCCACTGGCCCCT

mAb-hERG1 VL pCRBlunt-SEQU. N°1

GACATTGTGCTCACACAAACTCCACTCACTTTGTCGGTTAACATTGGTCAACCAGCCTCTATCTCTTGCAAGTCAAGTCAGAGC
CTCTTATATACTAATGGAAAAACCTATTTTAATTGGTTATTACAGAGGCCAGGCCAGTCTCCAAAGCGCCTAATCTATCTGGTGTC
TAAACTGGACTCTGGAGTCCCTGACAGGTTCACTGGCAGTGGATCAGGAACAGATTTTACACTGAAAATCAGCAGAGTGGAGG
CTGAAGATTTGGGAGTTTATTACTGCGCGCAAGGTACACATTTTCCGTGGACGTTCGGTGGAGGGACCAAGCTGGAAATCAAA
CGGGCTGATGCTGCACCAACTGTATCCA

Supplementary Figure 11: Five representative pCR-Blunt-V_H and one representative pCR-Blunt-V_L nucleotidic sequences obtained from bacterial DNA extraction. Highlighted in yellow nucleotide sequences corresponding to the triplet corresponding to the Phe aa.

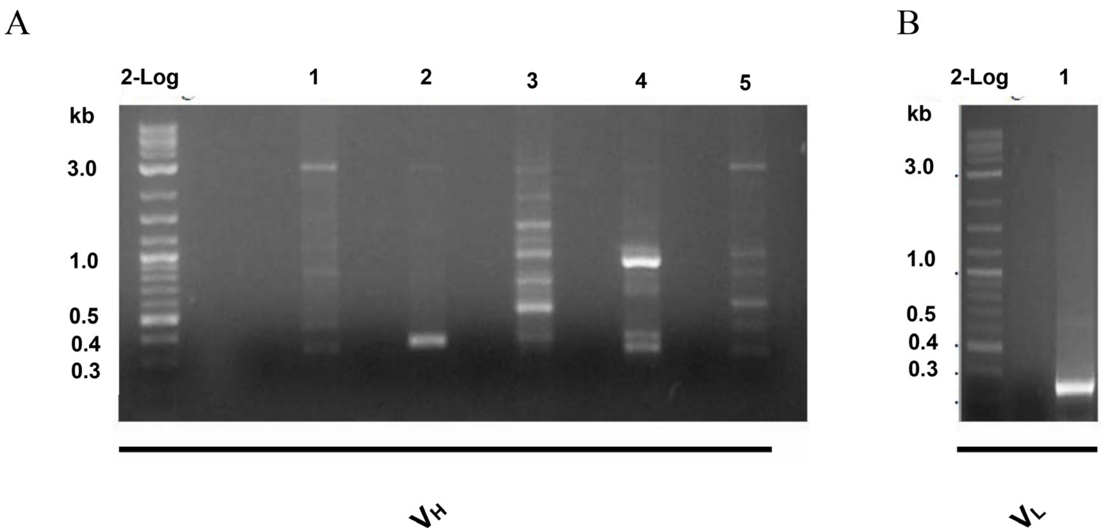

Supplementary Figure 12: Isolation of V_H and V_L regions through PCR amplification. (**A**) Amplification of V_H gene with different combination of primers: IgG2b_revdegH2_ for, which successfully amplified V_H chain is reported in lane 2. 2-Log DNA Ladder is on the left. (**B**) Amplification of V_L gene through degL(κ)_for- κ_rev primers. 2-Log DNA Ladder is on the left.

TTGTTATTACTCGCGGCCCAGCCGGCC**ATG**GCCCAGGTGCAGCTGCAGGTCGACGAGGTCCAACTGCAACAGTCTGGACCTGAACTGG
TGAAGCCTGGGGCTTCTGTGAAGATATCCTGCAAGACTTCAGGATACACATTCACTGAATACACCGTTCACTGGGTGAAACAGAGCCA
TGGAAAGAGCCTTGAATGGATTGGAGGCATTAATCCTAATGGTGGTACTACCTATAATCAGAAGTTCAAGGGCAAGGCCACATTGACT
ATTGACAAGTCCTCCAGCTCAGCCTTCATGGAGCTCCGCAGCCTGACATCTGAGGATTCTGCAGTCTATTACTTTGCAACAGGTTGGG
GACCTGACTACTGGGGCCAAGGCACCACTCTCACAGTCTCCTCAGCCAAAACAACACCCCCATCAGTCTATCCACTGGCCCCTGGCTC
GAGT**GGTGGAGGCGGTTCAGGCGGAGGTGGCTCTGGCGGTAGTGCA**CTGGATATTGTGCTGACACAATCTCCACTCACTTTGTCGGTT
AACATTGGTCAACCAGCCTCTATCTCTTGCAAGTCAAGTCAGAGCCTCTTATATACTAATGGAAAAACCTATTTTAATTGGTTATTAC
AGAGGCCAGGCCAGTCTCCAAAGCGCCTAATCTATCTGGTGTCTAAACTGGACTCTGGAGTCCCTGACAGGTTCACTGGCAGTGGATC
AGGAACAGATTTTACACTGAAAATCAGCAGAGTGGAGGCTGAAGATTTGGGAGTTTATTACTGCGCGCAAGGTACACATTTTCCGTGG
ACGTTCGGTGGAGGGACCAAGCTGGAAATCAAACGGGCTGATGCTGCACCAACTGTATCCGCGGCCGCAGAACAAAAACTCATCTCAG
AAGAGGATCTGAATGGGGCCGCA**TAG**A

Supplementary Figure 13: Sequencing results obtained from the only positive colony obtained pHenIx vector showing the proper orientation VH-linker-VL.

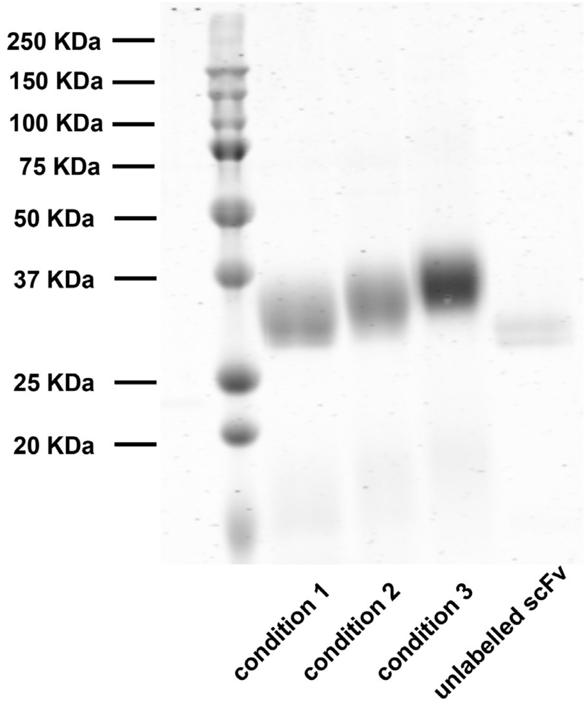

Supplementary Figure 14: SDS PAGE showing scFv-hERG1-Cys antibody after conjugation with Alexa750 fluorophore. D.O.L. for each condition resulted to be 1.48 (condition 1), 2.17 (condition 2) and 4.25 (condition 3), band in the fourth line corresponds to the unlabelled antibody. One aliquot of proteins from each labelling condition was controlled on 15% SDS PAGE confirming the data obtained by D.O.L. calculation: for condition 1 is evident an increase of molecular weight of protein of 1300 g/mol (dye molecular weight), for condition 2 an increase 2600 g/mol and and for condition 3 and increase of 5200 g/mol.
